# Comprehensive Protocol for the Identification and Characterization of New Psychoactive Substances in the Service of Law Enforcement Agencies

**DOI:** 10.3389/fchem.2020.00693

**Published:** 2020-09-25

**Authors:** Ewa Bulska, Robert Bachliński, Michał K. Cyrański, Magdalena Michalska-Kacymirow, Wioletta Kośnik, Paweł Małecki, Karol Grela, Michał A. Dobrowolski

**Affiliations:** ^1^Faculty of Chemistry, Biological and Chemical Research Center, University of Warsaw, Warsaw, Poland; ^2^Central Forensic Laboratory of the Police, Warsaw, Poland

**Keywords:** new psychoactive substance (NPS), synthetic cannabinoid (SC), X-ray diffraction, ES Q/TOF MS/MS, LC-Q/TOF

## Abstract

A non-routine, comprehensive protocol for characterization of emerging new psychoactive substances (NPS) including chemical structures, impurities, as well as crystal structures, has been developed to facilitate the work of law enforcement agencies. A set of NPS has been synthesized, identified, and characterized by various analytical methods in order to be used as certified reference standards (CRMs). Seven selected compounds (5-IT, NM-2201, MT-45, AB-CHMINACA, UR-144, 5F-PB-22, and 4-CMC) were synthesized on the laboratory scale, then the process was upscaled to semi-technical. All products were analyzed by electrospray Q/TOF-MS/MS for molecular structure identification. The presence of by-products, as well as metal impurities, arising from the performed syntheses, were characterized by reversed phase liquid chromatography (RP-HPLC) with DAD and Q/TOF-MS detection and inductively-coupled plasma with quadrupole mass spectrometer (ICP-QMS), respectively. Additionally, the crystal structures of UR-144, NM-2201, 5F-PB-22, and 4-CMC have been determined by single-crystal and powder X-ray diffraction.

## Introduction

New psychoactive substances (NPS) are a large group of chemical compounds that have been available on the world market since the beginning of the twenty-first century^1^,^2^. They differ in composition, but have one common feature: they affect the central nervous system (CNS) of a human in a similar manner to previously known “classical” drugs. These substances are both synthetic and obtained from plants. They are known by many names: designer drugs, legal highs, bath salts, party pills, etc.

According to their chemical structures, they can be divided into several main groups: synthetic cannabinoids, cathinones, phenylethylamines, tryptamines, benzodiazepines, piperidines, pyrrolidines, and opioids. The majority of new drugs on the market in Poland (and throughout Europe)^1^ are synthetic cannabinoids, introduced as a legal alternative to marijuana^3^, and cathinones, legal substitutes for amphetamines (DeRuiter et al., 1994).

The interest in synthesizing cannabinoids began with the synthesis of dl-Δ^1^-tetrahydrocannabinol in 1965 (Mechoulam and Gaoni, 1965). Later, several research groups and commercial companies focused on synthesizing compounds with cannabimimetic activity. At first, only classical cannabinoids [e.g., HU-210 (Howlett et al., 1990) and Levonantradol (Koe, 1981)] were obtained. Non-classical cannabinoids [e.g., CP-47,497 and CP-59,540 (Melvin et al., 1984; Compton et al., 1992)] and AAIs (Huffman et al., 1994) were developed at the end of the twenty-first century after the so-called cannabinoid receptors CB1 (Matsuda et al., 1990) and CB2 (Munro et al., 1993) were identified and cloned. Since then, scientific interest in these compounds has increased leading to the synthesis of over 100 substances with high or medium affinity to the CB1 receptor^3^.

Differently substituted *n*-alkyl indole or indazole-3-carbonyl derivatives are distributed around the world as synthetic cannabinoids. They appeared on the market in Europe around 2006 and were first detected toward the end of 2008. In 2018 a further 11 new synthetic cannabinoids were reported for the first time, bringing the total number reported to the EU Early Warning System to 190 (EU Drug Markets Report, 2019). This makes the synthetic cannabinoids the largest group of substances monitored by the EMCDDA, and reflects the overall demand for cannabis within Europe and the rapid pace by which manufacturers can produce and supply new cannabinoids to circumvent drug laws, and similar reports are coming from the other parts of the world^4^ The race between illegal laboratories and law enforcement agencies is never-ending. Newly substituted compounds appear on the drug market just after their predecessors fall under regulation, meaning there is an essential need to develop rapid, effective, and easy to apply scientific techniques for the purposes of law enforcement.

Liquid chromatography combined with mass spectrometry (LC/MS) and tandem mass spectrometry (LC/MS/MS) has become a powerful tool for the identification and characterization of psychoactive substances as well as degradants, metabolites, and process impurities (Eckers et al., 1997). Among the many methods available, particularly noteworthy is liquid chromatography coupled to high-resolution quadrupole time-of-flight mass spectrometry (LC-Q/TOF) (Fornal et al., 2013). This technique enables the tentative identification of an unknown compound based on the prediction of its chemical formula from accurate ion mass measurement and characteristic isotopic pattern (Aszyk and Kot-Wasik, 2016). This technique is characterized by high sensitivity, selectivity, and versatility. Moreover, it is considered a very great tool for the identification of species and can be very useful when reference standards are not available. Additionally, the sample amount needed for the analysis is very small, and sample preparation is straightforward—it is just dissolved and analyzed (Eckers et al., 1997; Lee et al., 2009). The analytical protocol developed by this study was successfully used for the identification of various metabolites with a view to doping control (Kwiatkowska et al., 2018; Grucza et al., 2019). Modern LC-Q/TOF mass spectrometers offer high chromatographic and mass resolutions and high mass accuracy measurements of both parent and fragment ions and provide sufficient information to detect and identify compounds, even in complex matrices (Eckers et al., 1997). In this work, we used the high potential of LC-Q/TOF mass spectrometry for the effective identification of new psychoactive substances in our research.

The X-ray diffraction technique is not commonly used in NPS analysis, especially synthetic cannabinoids. This is mainly due to their physical form (plant or resin) found on the worldwide black market. Here we present crystal structures and diffraction patterns of three synthetic cannabinoids: UR-144, NM-2201, and 5F-PB-22; obtained by single-crystal and powder X-ray diffraction. The crystal structure of UR-144 was reported before (Banister et al., 2015) but has not been deposited in the Cambridge Structural Database (CSD) (Groom et al., 2016). We redetermined it at a lower temperature (sometimes a possible reason for phase transitions) and deposited all the structures in the CSD before publishing this article.

Only a few articles reporting crystal structures of synthetic cannabinoids have been published [e.g., (Hongfeng et al., 2005; Huffman et al., 2005; Nycz et al., 2010; Hong et al., 2011; Banister et al., 2013)]. There is a large need to develop the CSD further for new psychoactive substances. It may serve as a very quick and reliable source for identifying illicit substances, as it is a common tool, especially for the people working in the government laboratories.

In this article, the authors describe a carefully developed non-routine analytical protocol toward the comprehensive characterization of NPS. Selected physicochemical techniques make it possible to obtain the complementary information of NPS, namely those considered as emerging substances found on the illegal drug market. Completely characterized compounds could serve as reference substances to facilitate the work of law enforcement agencies. Thus, the combination of analytical instrumental techniques and X-ray diffraction may allow for rapid and credible studies of evidence collected at the crime scene. Moreover, such protocols could enable forensic scientists to gather supporting information that may be used for judicial purposes as incontestable proof.

## Materials and Methods

### Synthesis of Compounds

**Figure 1:**
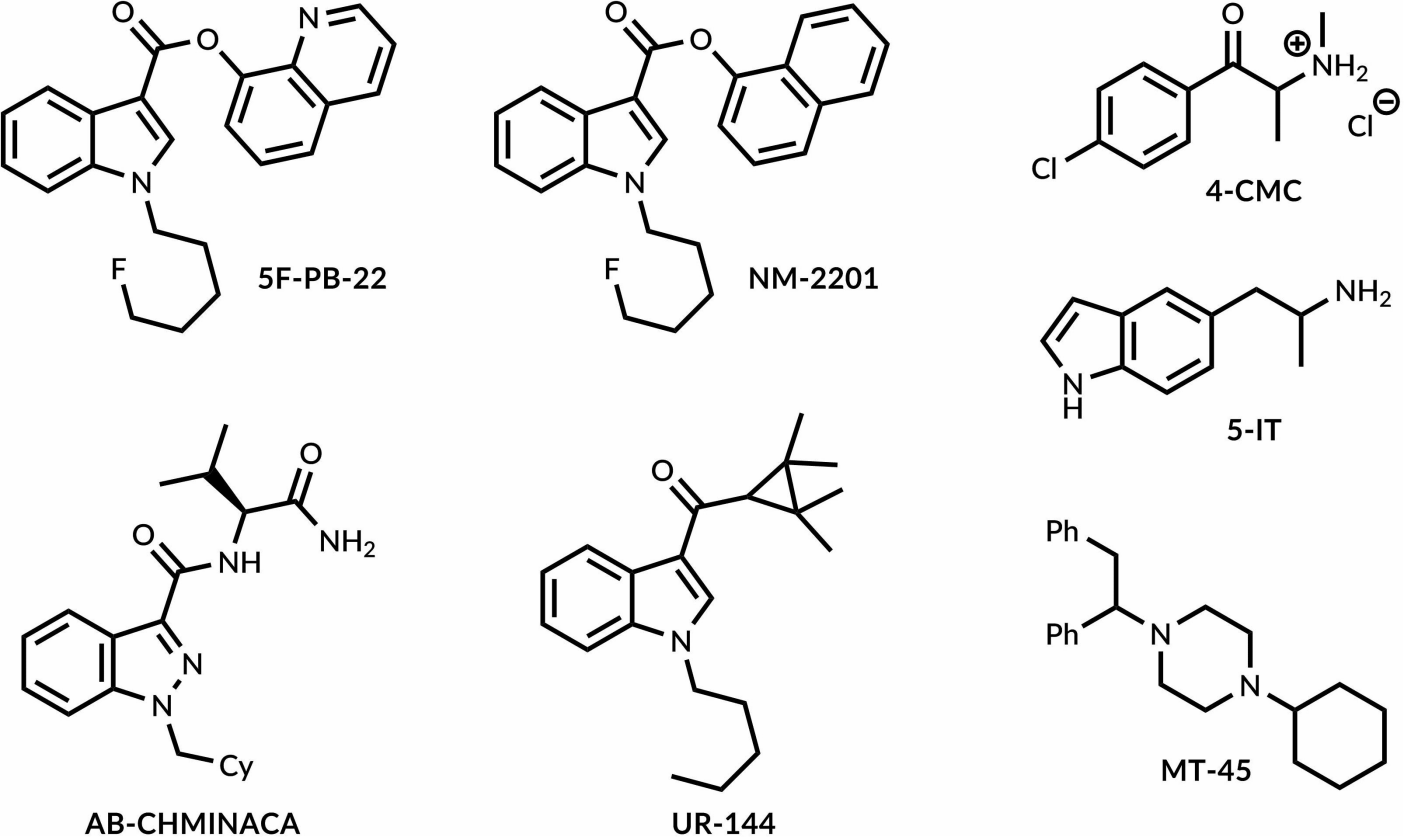
Designer drugs synthesized in this study.

We have synthesized seven NPS widely circulating on worldwide black markets^1^,^2^ and belonging to 4 diverse groups: synthetic cannabinoids, cathinones, phenethylamine, and piperazine derivatives (Figure 1).

Syntheses of 5F-PB-22, NM-2201, and UR-144 were accomplished according to a modified Kassiou et al. procedure (Banister et al., 2015) (Supplementary Schemes S1, S2 in the Electronic Supplementary Material, ESI). The key intermediate—*N*-(5-fluoropentyl)-indole-3-carboxylic acid—was obtained from indole in three steps (*N*-alkylation, acylation with trifluoroacetic acid anhydride, and hydrolysis of the corresponding trifluoromethyl ketone) with 88% overall yield. Subsequent esterification led to 5F-PB-22 and NM-2201 in 91 and 72% yield, respectively. UR-144 was obtained in good 58% overall yield in two steps (*N*-alkylation and acylation with 2,2,3,3-tetramethylcyclopropanecarboxylic acid) from commercially available indole.

Synthesis of 4-CMC was accomplished according to a modified Bak et al. procedure (Bak et al., 2019) (Supplementary Scheme S3 in the ESI). The reaction was performed starting from commercially available 4-chloropropiophenone in a series of α-bromination and amination reactions and the desired product was obtained in a 68% overall yield.

Synthesis of MT-45 was performed according to a modified Umemoto et al. procedure (Natsuka et al., 1975) (Supplementary Scheme S4 in the ESI). Here, the sequence of oxazolidine formation, Grignard reaction, and amination led to MT-45 in 46% overall yield.

Synthesis of 5-IT employed a modified Szabo *et al*. procedure (Troxler et al., 1968) (Supplementary Scheme S5 in the ESI). Condensation of indole-5-carboxaldehyde with nitroethane, followed by lithium aluminum hydride reduction resulted in 5-IT in 75% overall yield.

Synthesis of AB-CHMINACA was accomplished according to a modified Kassiou et al. procedure (Longworth et al., 2016) (Supplementary Scheme S6 in the Supporting Information). Protection of 1*H*-Indazole-5-carboxylic acid as methyl ester, followed by alkylation with (bromomethyl)cyclohexane and deprotection allows obtaining a key intermediate, *N*-(cyclohexylmethyl)indazole-5-carboxylic acid. Subsequent amidation with valinamide hydrochloride resulted in AB-CHMINACA in 58% overall yield.

### Sample Description

The investigations were carried out for the selected NPS (UR-144, MT-45, 4-CMC, NM-2201, 5-IT, AB-CHMINACA, 5F-PB-22) synthesized on the laboratory scale as well as after upscaling to semi-technical. Groups of samples were named as “laboratory sample” (A) and “semi-technical' sample” (B).

### Sample Preparation for Chemical Analysis

Laboratory (A) and semi-technical (B) samples were prepared as follows: approximately 1 mg of the individual product (compound of interest) was dissolved in 1 ml of acetonitrile (ACN), followed by a series of dilutions in ACN:H_2_O (1:1) solution, depending on the detection method used (MS; MS/MS; UV-Vis).

### Identification of Compounds After Synthesis

To identify the specific compounds, a main product of synthesis, the exact mass was measured and the parent ion was fragmented using a Q/TOF mass spectrometer (6540; Agilent) with direct sample introduction (syringe pump). The parameters of the mass spectrometer were as follows: ionization type: electrospray; ionization: positive ion mode; voltage on the capillary: 3,500 V; gas temperature: 300° C; drying gas flow: 8 L/min; nebulizer: 35 psi; shielding gas temperature: 350° C; shield gas flow: 11 L/min; fragmentor voltage: depending on the compound 100 or 175 V; voltage on the collecting cone: 65 V; collision energy: 10, 20 and 40; mass range for MS scan mode: 100–950 m/z; mass range for MS/MS mode: 40–600 m/z.

### Melting Points

Melting points were determined for UR-144, NM-2201, and 5F-PB-22 using a Mettler Toledo MP70 Melting Point System. They were 73.5, 122, and 87°, respectively.

### Organic Impurities

It is essential to identify the impurities arising from the synthesis process for any items that may serve as a certified reference standard. To separate the main compounds and their potential impurities, reversed phase high-performance liquid chromatography (RP-HPLC) was used followed by a diode array detector (DAD) and a high-resolution mass spectrometer (Q/TOF-MS). An HPLC apparatus with a DAD detector (1290; Agilent) and a Q/TOF mass spectrometer (6540; Agilent) was used for that purpose. Chromatograms with DAD detection were recorded at three wavelengths (254, 230, and 295 nm). Tables 1, 2 present the parameters of the chromatographic separation, detectors, and gradient elution program. Table 3 presents the parameters of the mass spectrometer.

**Table 1:** HPLC setup parameters.

Solvent A	Ammonium formate 1 g/L in 10% ACN
Solvent B	Ammonium formate 1 g/L in 90% ACN
Column	LumiSep C18, 100 x 2.1, 3.0 μm
Velocity of mobile phase	0.5 mL/min
Injection	2.00 μL
Column temperature	35^°^ C
Detector	DAD: 254 nm, 230 nm and 295 nm; ESI-MS-Q/TOF

**Table 2:** Gradient elution program for HPLC separation.

**Time [min]**	**% A**	**% B**
0.0	100	0
3.00	100	0
8.00	0	100
8.01	100	0
15.00	100	0

**Table 3:** Experimental parameters for the MS/MS identification experiments.

Ionization: electrospray
Mode of ionization: positive
Voltage on capillary: 3,500 V
Gas temperature (1): 300^°^ C
Gas flow (1): 8 L/min
Nebuliser pressure: 35 psi
Gas temperature (2): 350^°^ C
Gas flow (2): 11 L/min
Voltage on fragmentor: 175 V
Voltage on skimmer: 65 V
Collision energy: 10, 20, and 40
Mass range for MS scan mode: 100–950 m/z
Mass range for MSMS mode: 40–600 m/z

### Inorganic Impurities

To assess the elemental impurities, which can be introduced during the synthesis of all investigated products, ICP-QMS (NexIon 300D, Perkin-Elmer) was used. All compounds were subjected to microwave-assisted mineralization (Milestone MultiWave) before measurements. The validation of the analytical procedure was performed with the use of tetracycline and amoxicillin pure compounds as well as after the addition of known amounts of multi-element standards. Table 4 presents the parameters of the ICP-MS conditions.

**Table 4:** Optimal measurement conditions for elemental analysis by ICP-MS.

**Parameter**	**Description**
Spray chamber	Scott, Quartz
Atomizer	Coaxial (Mainhardt)
Plasma torch	Quartz
Sampling cone	Nickel
The forming cone	Nickel
Frequency of the generator	40 MHz
Resolution of mass signals	(0.7 ± 0.05) a.m.u.
Generator power	1,000 W
Deflector voltage	−9 V
Voltage on the analog detector	−1,800 V
Voltage on the pulse detector	950 V
Plasma gas flow	15.00 L/min
Auxiliary gas flow	1.2 L/min
The flow of atomizing gas	0.89 L/min
Stop time	50 ms
The number of sweeps	20
Number of repetitions	3-5
Solution dispensing speed	1 mL/min

### Compound Characteristics

As indicated above, all the compounds were synthesized at the University of Warsaw. We were able to obtain crystals of UR-144 (**1**), NM-2201 (**2**), 5F-PB-22 (**3**), and 4-CMC suitable for X-ray experiments by slow evaporation from ethanol. Single crystals were measured at 130 (2) K on a Bruker D8 Venture Photon100 diffractometer with a TRIUMPH monochromator and MoK_α_ fine-focus sealed tube (λ = 0.71073 ). The structure of 4-CMC has been deposited earlier at ambient conditions (Nycz et al., 2016). We redetermined it at low temperature (to check for possible phase transitions) but, as we would like to focus on crystal structures of synthetic cannabinoids in this article, for details please refer to the Electronic Supplementary Information (ESI).

The crystals were positioned 40, 50, and 100 mm from the CCD camera for (**1**), (**2**), and (**3**), respectively. A total of 1,613, 340, and 1,101 frames were measured with exposure times of 8.96, 0.28, and 0.31 h for (**1**), (**2**), and (**3**). Data were collected using the APEX2 program (APEX2, 2013), integrated with the Bruker SAINT software package (SAINT, 2013), and corrected for absorption effects using the multi-scan method (SADABS) (SADABS, 2012). The structures were solved and refined using the SHELX Software Package (Sheldrick, 2015) with the atomic scattering factors taken from the International Tables (International Tables for Crystallography, 1992). Crystal data and structure refinement are specified in Table 5. The structure of (**3**) is disordered with a fluorine atom at two alternative sites with 75–25% occupancies. The figures were generated with Mercury (ver. 4.2.0) (Macrae et al., 2006).

**Table 5:** Crystal data and structure refinement for: UR-144 (1), NM-2201 (2), 5F-PB-22 (3).

**Compound**	**(1)**	**(2)**	**(3)**
Empirical formula	C_21_H_29_N_1_O_1_	C_24_H_22_F_1_N_1_O_2_	C_23_H_21_F_1_N_2_O_2_
Formula weight	311.45	375.43	376.43
Temperature [K]	130	130	130
Space group	*P2_*1*_/n*	*P2_*1*_/c*	*P-1*
Unit cell dimensions			
*a* [Å]	12.0920(5)	12.0583(6)	8.388(5)
*b* [Å]	10.7612(4)	13.5389(8)	10.309(7)
*c* [Å]	13.9030(5)	11.4574(6)	11.853(6)
α [°]	90	90	68.75(3)
β [°]	93.743(2)	95.100(2)	86.52(2)
γ [°]	90	90	79.24(3)
Volume *V* [Å^3^]	1805.26(12)	1863.09(17)	938.5(9)
Z [molecules/cell]	4	4	2
*D*_calculated_ [g cm^−3^]	1.146	1.338	1.332
Absorption coefficient μ/ mm^−1^	0.069	0.091	0.092
θ range for data collection [°]	2.31-25.00	2.27-25.00	2.15-25.50
Limiting indices	−14 ≤ *h* ≥ 14	−14 ≤ *h* ≥ 14	−10 ≤ *h* ≥ 10
	−12 ≤ *k* ≥ 12	−16 ≤ *k* ≥ 16	−12 ≤ *k* ≥ 12
	−16 ≤ *l* ≥ 16	−13 ≤ *l* ≥ 13	−14 ≤ *l* ≥ 14
Reflections collected/unique	25,423/3,170	21,433/3,279	27,748/3,491
Data/parameters	3,170/214	3,279/254	3,491/348
Goodness of Fit	1.047	1.152	1.125
Final *R* index (*I* > 2σ)	0.0348	0.0401	0.0390
w*R*^2^	0.0830	0.0803	0.0855
Largest diff. Peak and hole [Å^−3^]	0.232 and −0.184	0.212 and −0.192	0.248 and −0.267
CCDC	1842294	1842293	1842292

CCDC 1842292-1842294, 1943878 contains the supplementary crystallographic data for this paper. These data can be obtained free of charge via http://www.ccdc.cam.ac.uk/conts/retrieving.html.

Powder X-ray diffraction measurements were performed at room temperature on a Bruker D8 Discover diffractometer (CuK_α1_ and CuK_α2_ radiation in a *ca*. 2:1 intensity ratio). The samples were placed in sealed quartz capillaries (0.5 mm diameter).

## Results and Discussion

### Identification of Products of Synthesis

**Figure 2:**
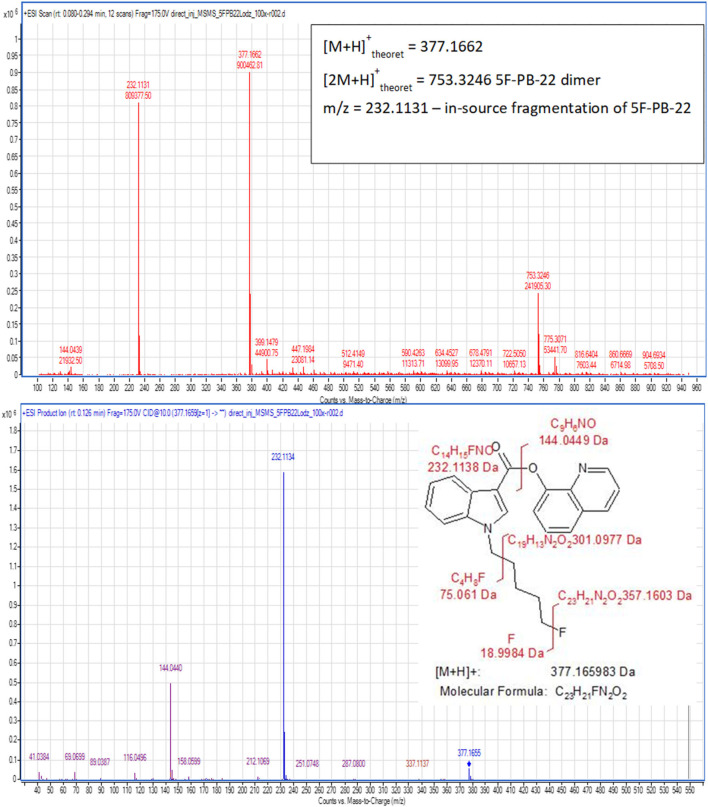
Mass spectra for 5F-PB-22 (B).

The first approach was to perform the identification of all synthesized compounds obtained on both the laboratory and semi-technical scales. This was done according to the common, validated scheme, exemplified by the 5F-PB-22. The mass spectrum (MS) and fragmentation spectrum (MS/MS) are presented along with the proposed fragmentation path in Figure 2, for 5F-PB-22 obtained on the semi-technical scale (B). The careful evaluation of all spectra indicates the presence of a pseudomolecular ion [M+H]^+^ at 377.1660 m/z units, dimer of pseudomolecular ion [2M+H]^+^ at 753.3247 m/z units and the product of in-source fragmentation of the pseudmolecuar ion with m/z 232.1130 units. Below, the product ion spectra of the pseudomolecular ion [M+H]^+^ (377.1660 m/z), acquired at a collision energy of 10 eV, is also shown with its proposed fragmentation path. The information gained from these ion product spectra confirms that the compound is 5F-PB-22.

Mass spectra for other compounds obtained on the semi-technical scale are presented in the supplementary material as follows: 5-IT (B)—Supplementary Figure 18, NM-2201 (B)—Supplementary Figure 19, AB-CHMINACA (B)—Supplementary Figure 20, and UR-144 (B)—Supplementary Figure 21.

To deduce the mass of the compound of interest from the mass spectra registered at MS/MS, the accuracy of measurements was established (Table 6). The mass accuracy (MA), expressed as part per millions (ppm) was calculated as follows:

**Table 6:** The accuracy of measurements established for compounds obtained on the semi-technical scale.

**Compound**	**Formula**	**M_**THEORET**_ (calculated m/z [M+H])**	**M_**EXP**_ (measured m/z [M+H])**	**MA (mass accuracy) [ppm]**
5F-PB-22 (B)	C_23_H_21_FN_2_O_2_	377.1659	377.1662	0.8
5-IT (B)	C_11_H_14_N_2_	175.1230	175.1227	1.7
NM-2201 (B)	C_24_H_22_FNO_2_	376.1707	376.1682	6.6
AB-CHMINACA (B)	C_20_H_28_N_4_O_2_	357.2285	357.2295	2.8
UR-144 (B)	C_21_H_29_NO	312.2322	312.2323	0.3

MA = (M_exp_–M_theoret_)/M_theoret_ × 10^6^

The results indicate that the accuracy of the mass measurements is high and enables satisfactory identification of the compounds of interest.

Finally, the evaluation of the mass spectra, toward the identification of the specific compound was performed for all investigated products of synthesis. Then, those products that were identified as expected underwent further characterization for their organic and inorganic impurities as well as crystal structures. This was considered necessary, to use them as standard compounds for judicial purposes.

### Organic Impurities

**Figure 3:**
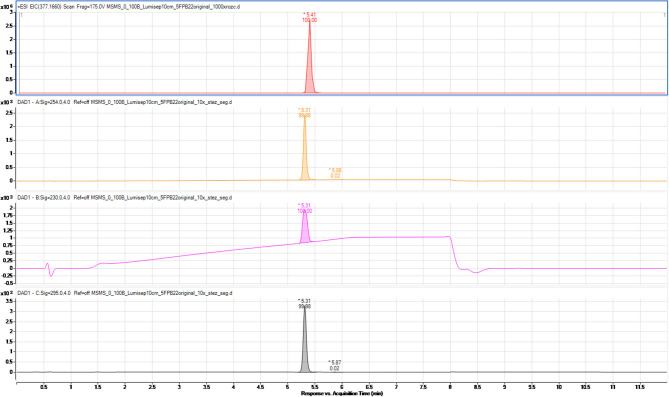
Chromatogram of 5F-PB-22 synthesized on the laboratory scale (A). MS detection (extracted ion chromatogram) and UV-Vis at three wavelengths (254, 230, and 295 nm).

**Figure 4:**
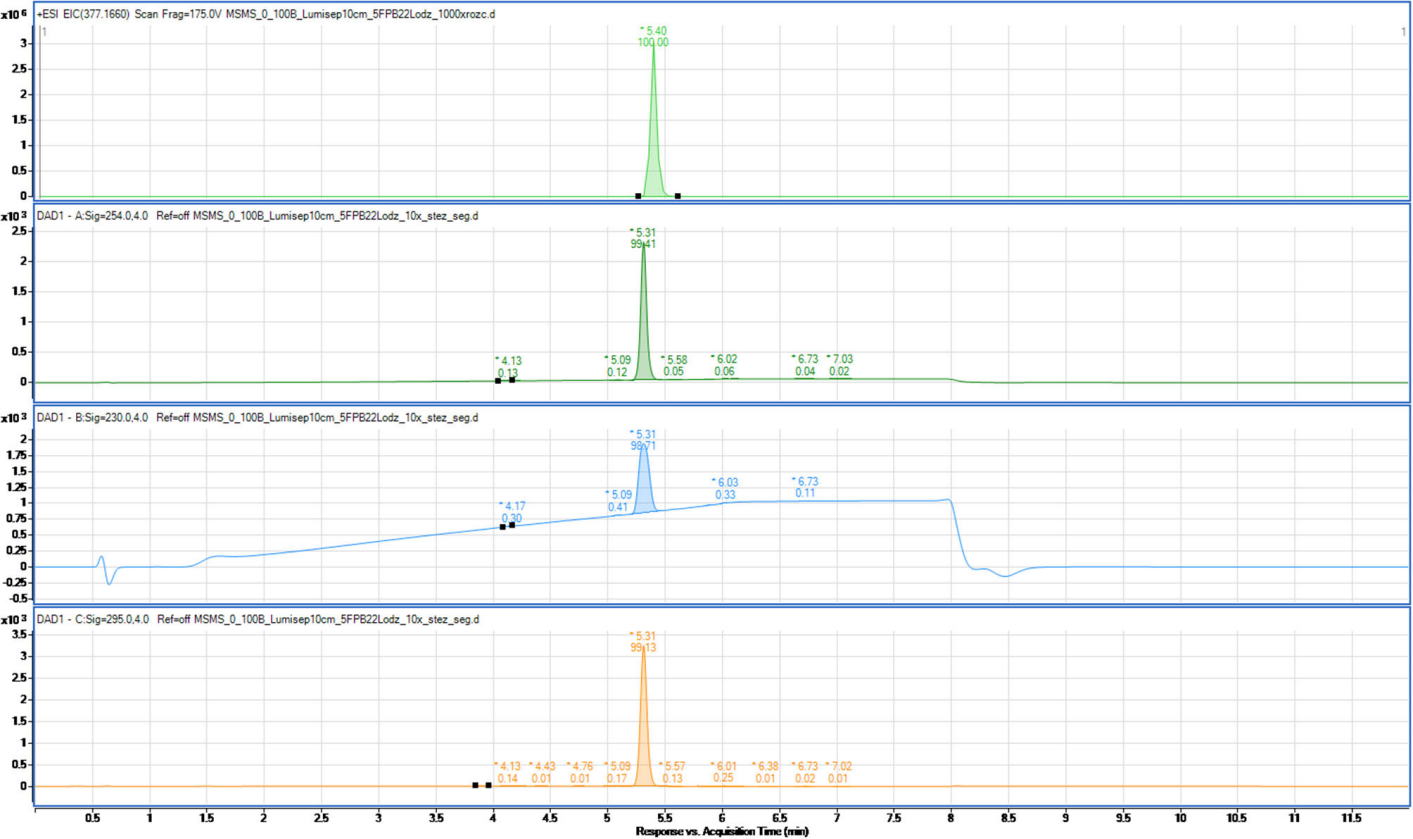
Chromatogram of 5F-PB-22 synthesized on the semi-technical scale (B). MS detection (extracted ion chromatogram) and UV-Vis at three wavelengths (254, 230, and 295 nm).

The composition of synthesized products of psychoactive substances was also examined to determine possible organic impurities, which could be the by-products of the synthesis processes on both laboratory and semi-technical scales. For this purpose, a procedure for the separation of the tested compounds (main products of synthesis) and their potential impurities has been developed using liquid chromatography coupled with a high-resolution mass spectrometer (Q/TOF-MS). To exemplify the process, chromatograms of 5F-PB-22 on the laboratory (A) and semi-technical scales (B) are shown in Figures 3, 4.

The other chromatograms obtained with the use of optimized separation parameters presented in Tables 1–3 and the UV spectra of the individual compounds are presented in the ESI (Supplementary Figures 22–28).

The results show that the products of synthesis performed either on the laboratory or semi-technical scale are high-purity organic compounds. Those results prove that they can be considered as high-purity reference materials.

### Inorganic Impurities

Although the organic impurities are the most important aspects considered for standard compounds with defined purity, in the course of this work it was essential to conduct the entire characterization of the obtained substances. For this purpose, the inorganic impurities, namely the elemental composition of the synthesis product were evaluated. The measurements were performed with ICP-MS and the following, most abundant isotopes were monitored: ^51^V, ^52^Cr, ^55^Mn, ^59^Co, ^60^Ni, ^63^Cu, ^66^Zn, ^88^Sr, ^95^Mo, ^101^Ru, ^105^Pd, ^107^Ag, ^111^Cd, ^137^Ba, ^195^Pt, ^205^Tl, ^208^Pb, ^202^Hg. The values of the limit of detection (LOD) and limit of quantification (LOQ) are listed in Table 7.

**Table 7:** The detection (LOD) and quantification (LOQ) limits for selected elements [mg/kg].

	**Pd**	**Pt**	**Ru**	**Zn**	**Cd**	**Co**	**Mn**	**Cu**	**Mo**
LOD	0.037	0.028	0.059	1.23	0.032	0.062	0.257	0.562	0.184
LOQ	0.066	0.040	0.099	1.36	0.056	0.107	0.318	0.677	0.235
	**Ni**	**Pb**	**Hg**	**V**	**Ba**	**Sr**	**Cr**	**Ag**	**Tl**
LOD	0.544	0.168	0.074	0.215	0.887	0.239	0.238	0.025	0.004
LOQ	0.885	0.210	0.115	0.258	0.937	0.267	0.340	0.044	0.006

The accuracy of the measurement condition was evaluated by the recovery either of spiked sample isotopes or of the elements measured in the presence of a defined matrix (tetracycline and amoxicillin solutions), which for all listed elements were no worse than 98 ± 5%.

All products of synthesis were examined for purities. Only those being characterized as pure with regard to organic impurities and those in which the content of listed elements was below the limit of detection (as presented in Table 7) were considered to be sufficient for crystallographic characterization.

To conclude, quantitative analysis of the elemental compositions indicated the high-purity of all synthesized substances, regardless of the scale of synthesis, which is essential to consider those substances as candidates for being Certified Reference Materials of established purity.

### X-ray Diffraction

**Figure 5:**
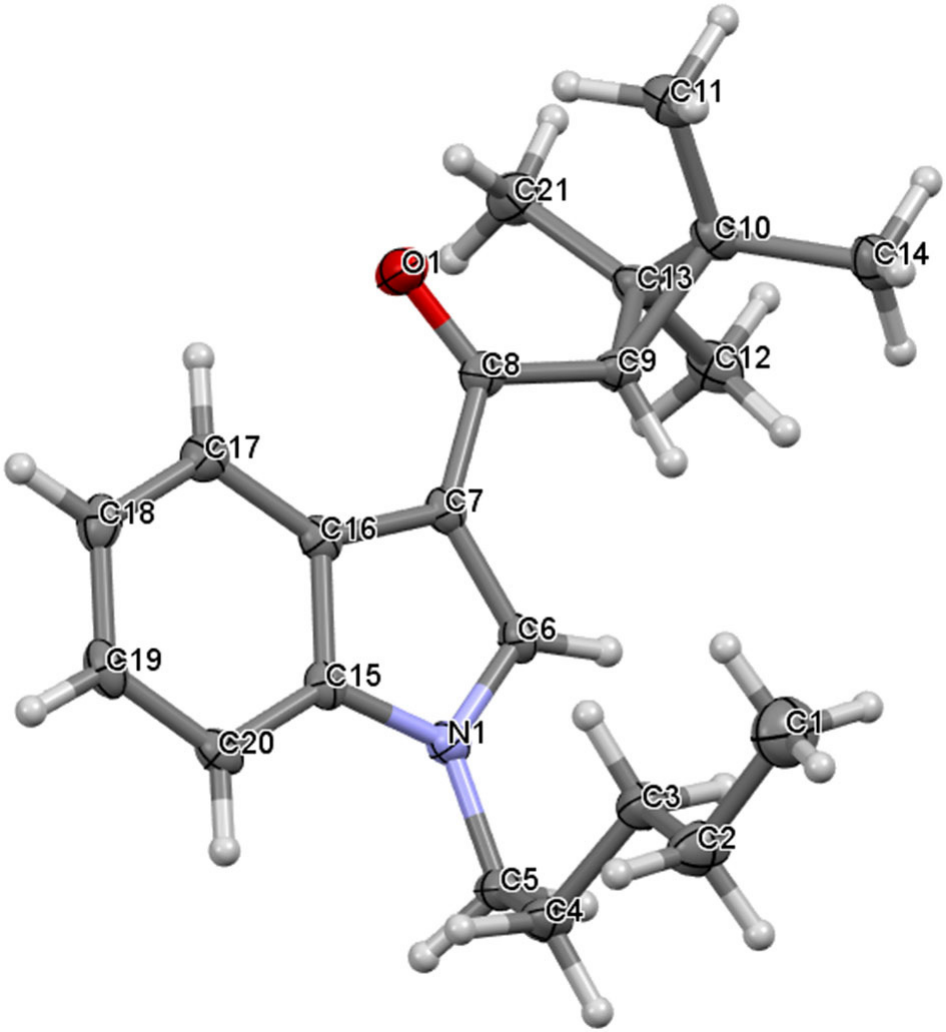
The molecular structure of UR-144 showing atom numbering and displacement ellipsoids at the 50% probability level.

**Figure 6:**
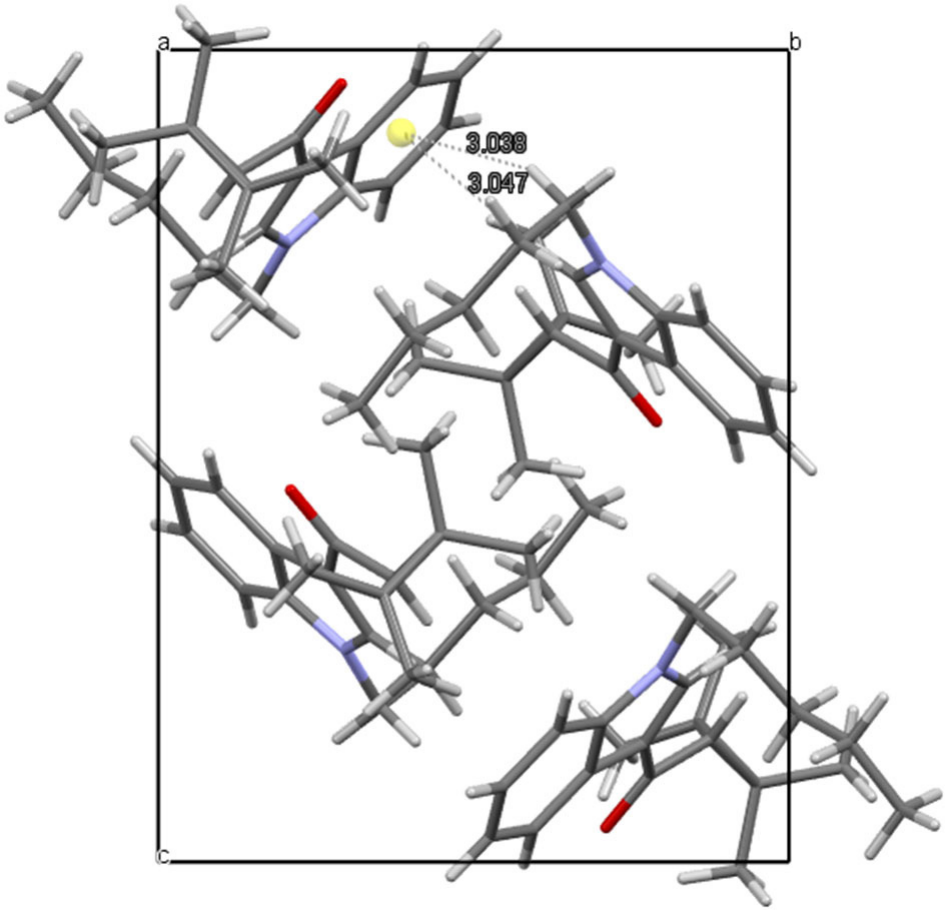
Crystal packing and short interactions in the crystal structure of UR-144. View along [100] direction.

(1-Pentyl-1H-indol-3-yl) (2,2,3,3-tetramethylcyclopropyl) methanone, UR-144 (**1**) crystallizes in a monoclinic system, space group P2_1_/n, with one molecule in an asymmetric part of the unit cell (Figure 5). The indole moiety is almost planar; the angle between the mean planes of the rings is 1.0°. The alkyl carbon chain is bent with respect to the nitrogen atom by 113.2° (C4-C5-N1). The structure is stabilized mostly by weak van der Waals interactions, but also weak C-H…π interactions in the crystal lattice. The latter involves the hydrogen atoms at C5 or C6 toward the centroid of the phenyl ring of the neighboring molecule (3.038 and 3.047 Å, respectively). The crystal packing and short interactions are shown in Figure 6.

**Figure 7:**
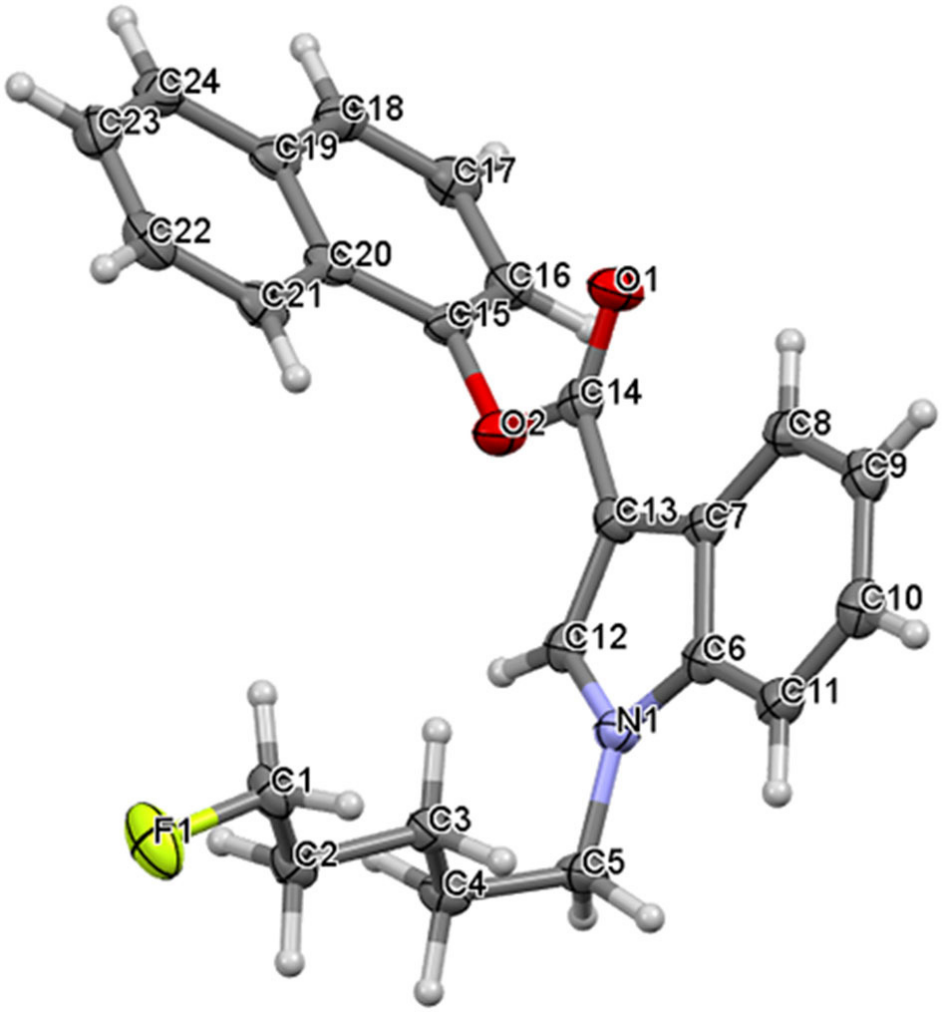
The molecular structure of NM-2201 showing atom numbering and displacement ellipsoids at the 50% probability level.

**Figure 8:**
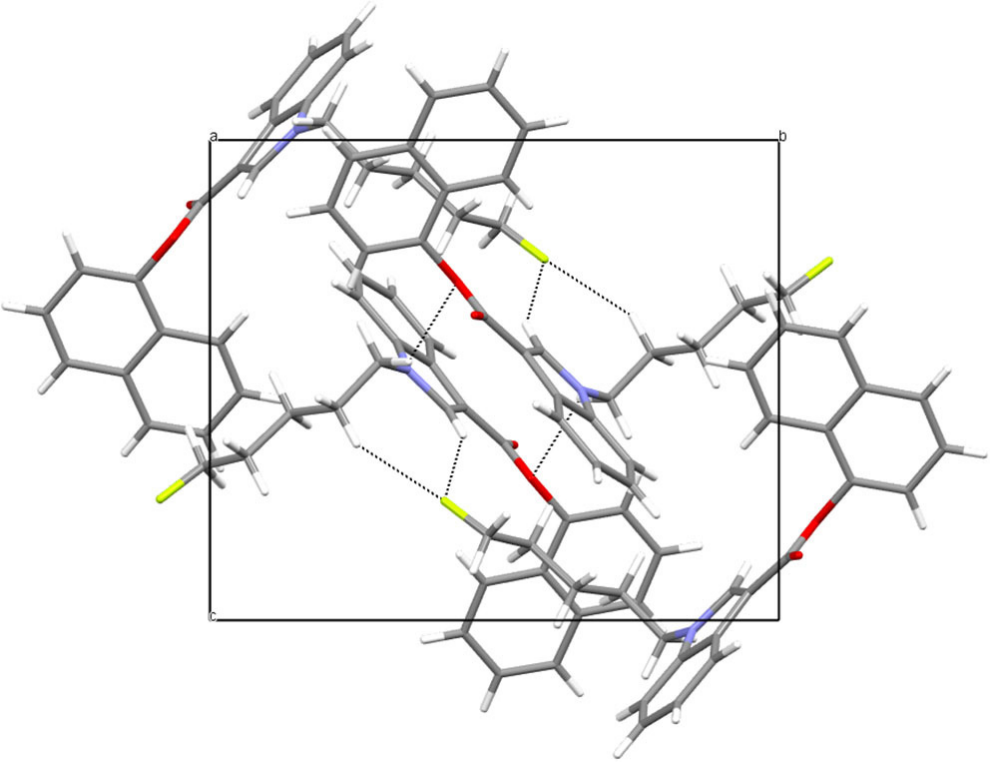
Crystal packing and short interactions in the crystal structure of NM-2201. View along [100] direction.

Naphthalen-1-yl 1-(5-fluoropentyl)-1H-indole-3-carboxylate, NM-2201 (**2**) crystallizes in a monoclinic system, space group P2_1_/c, with one molecule in an asymmetric part of the unit cell (Figure 7). The indole moiety is almost planar; the angle between the mean planes of the rings is 1.4°. The alkyl carbon chain is bent with respect to the nitrogen atom by 112.1° (C4-C5-N1). The structure is stabilized by weak C5-H…O2 hydrogen bonds (with the distance of 2.677Å), but also C-H…F interactions. For the latter, the interaction with the fluorine atom is bifurcated and the appropriate C4-H…F and C12-H…F distances equal to 2.502 and 2.574 Å, respectively. The crystal packing and short interactions are shown in Figure 8.

**Figure 9:**
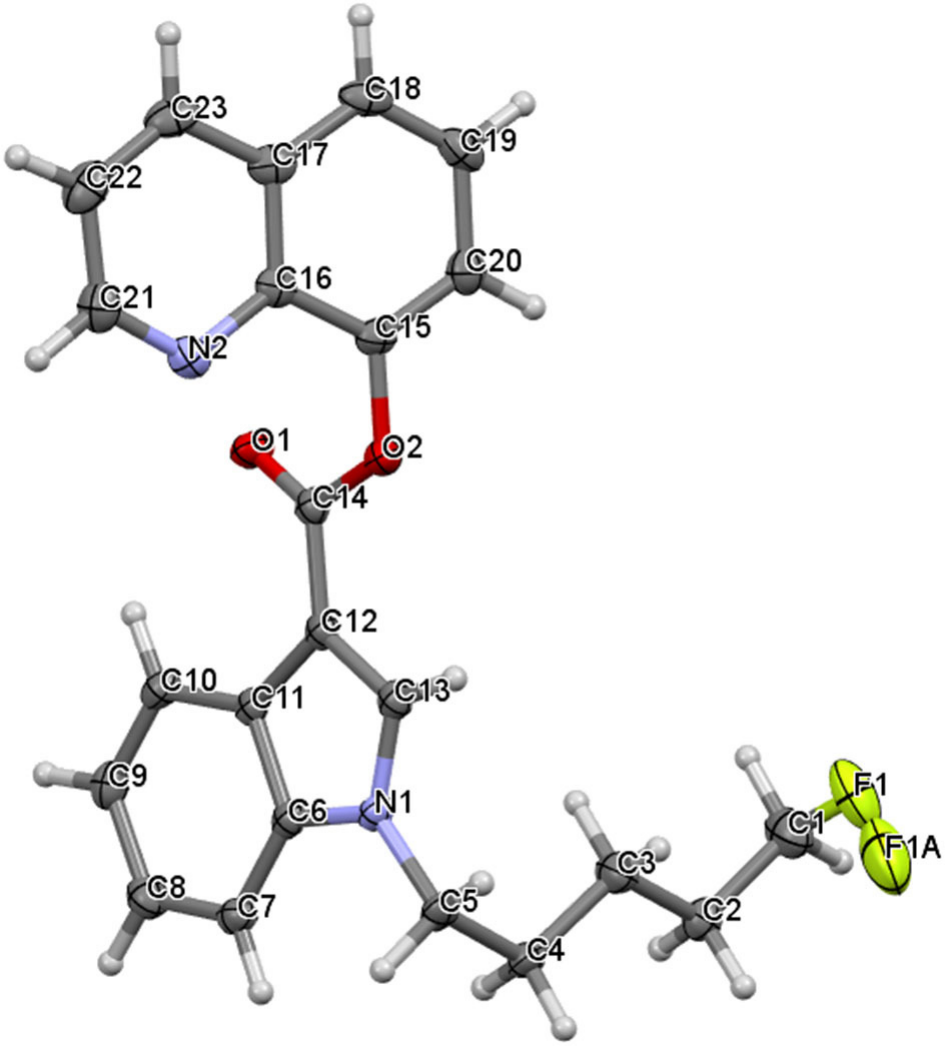
The molecular structure of 5F-PB-22 showing atom numbering and displacement ellipsoids at the 50% probability level. Fluorine atom at two alternative sites with 75–25% occupancy.

**Figure 10:**
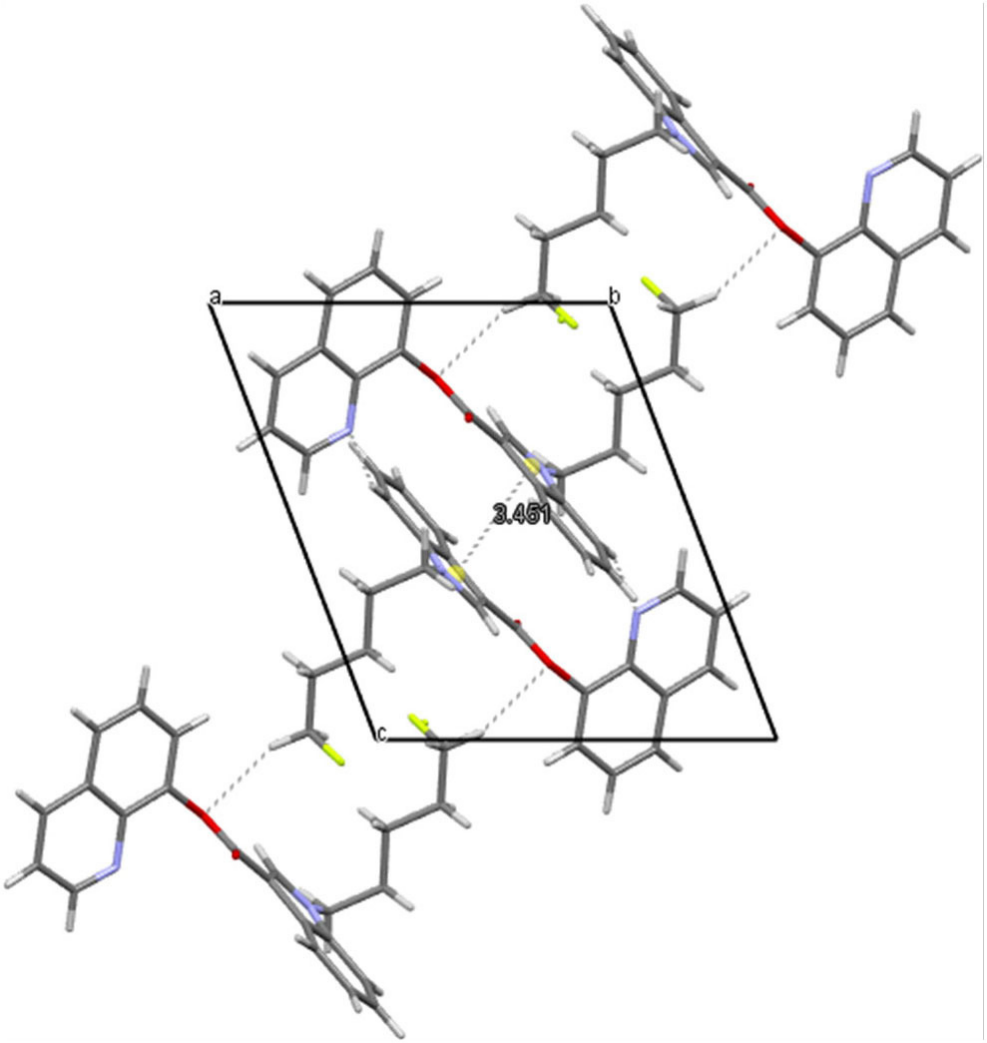
Crystal packing and short interactions in the crystal structure of 5F-PB-22. View along [100] direction.

Quinolin-8-yl 1-(5-fluoropentyl)-1H-indole-3-carboxylate, 5F-PB-22 (**3**) crystallizes in a triclinic system, space group P-1, with one molecule in an asymmetric part of the unit cell (Figure 9). The structure is disordered with a fluorine atom at two alternative sites with 75-25% occupancies. The indole moiety is almost planar; the angle between the mean planes of the rings is 2.0°. The alkyl carbon chain is bent with respect to the nitrogen atom by 113.2° (C4-C5-N1). The structure is stabilized by weak hydrogen bonds between the hydrogen at C7 and the nitrogen atom N2 (2.588 Å). Interestingly, the neighboring molecules form a kind of stack with overlapping pyrrole rings (distance between the centroids equal to 3.451 Å). The molecules also form weak hydrogen bonds between one of the hydrogens at C1 and the oxygen atom O2 (2.633 Å). Finally, there are C-H…F interactions in the crystal lattice involving the hydrogen atom at C23 of the quinoline moiety and the disordered fluorine atom of the neighboring molecule (2.645 and 2.574 Å, respectively). The crystal packing and short interactions are shown in Figure 10.

**Figure 11:**
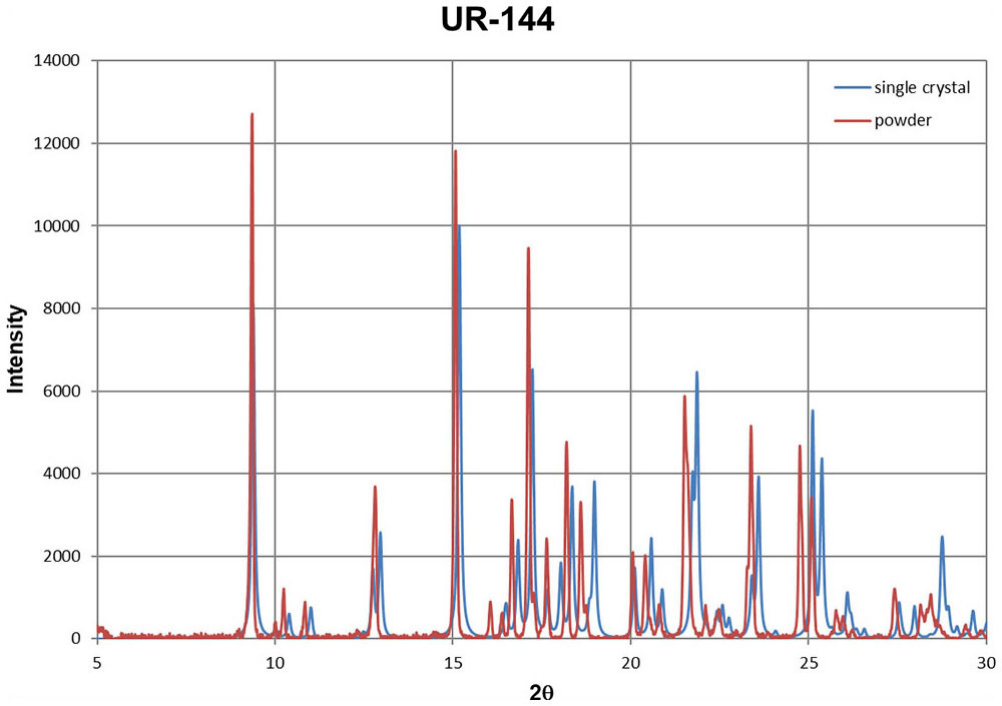
Powder diffraction patterns for UR-144, obtained from single crystal data (blue) and powder sample (red).

**Figure 12:**
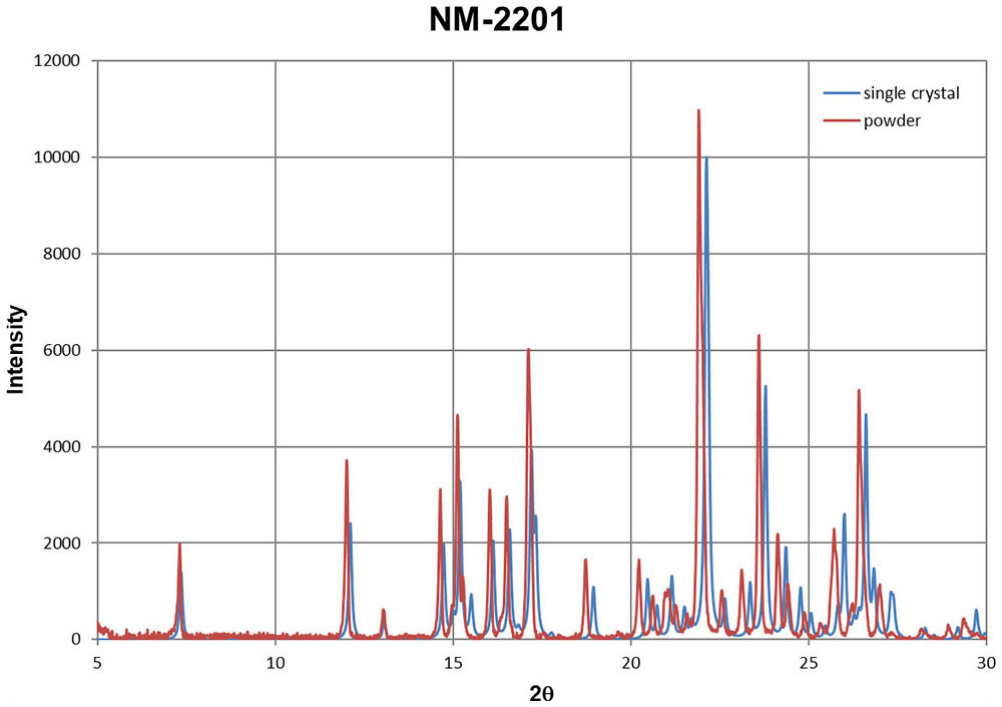
Powder diffraction patterns for NM-2201 obtained from single crystal data (blue) and powder sample (red).

**Figure 13:**
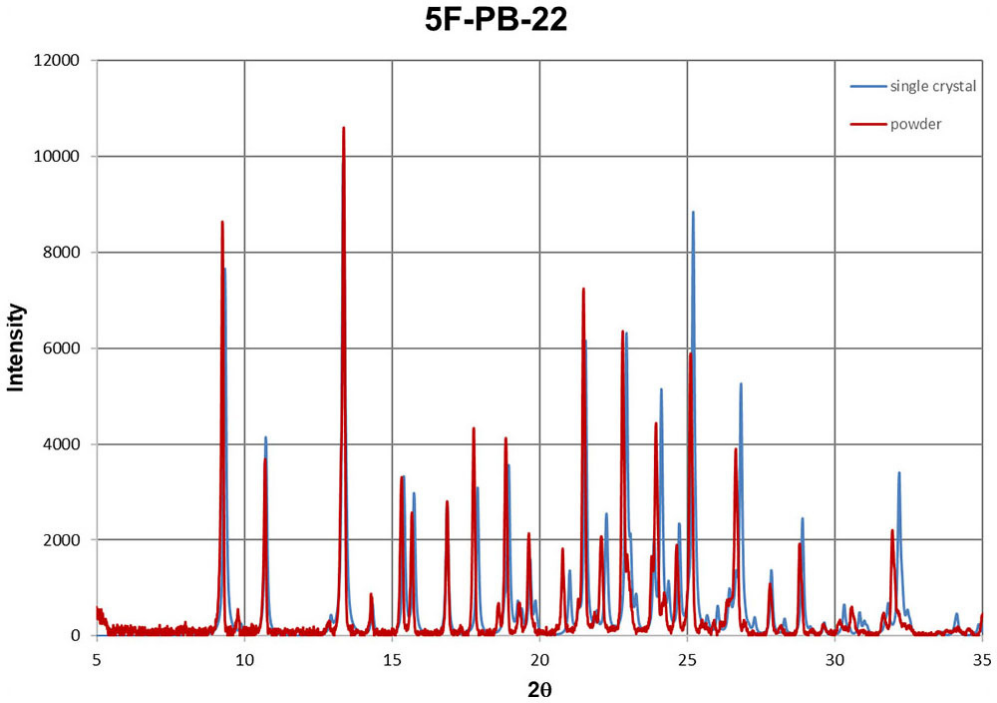
Powder diffraction patterns for 5F-PB-22 obtained from single crystal data (blue) and powder sample (red).

Additionally, the compounds (**1**), (**2**), and (**3**) have been measured by powder X-ray diffraction. It is a fast method to characterize the compound once we have solved its crystal structure (solving the crystal structure from powder diffraction data is possible, but not trivial and time consuming). Using generally available software it is straightforward to export powder diffraction patterns from the single crystal structure and compare it with an experimentally measured sample. The examples are shown for UR-144, NM-2201, and 5F-PB-22 in Figures 11–13. Despite the disorder in structure (**3**), the agreement in reflection positions for both powder patterns is very good. A small shift is caused by the different temperatures of the measurements (see the compound characteristic section). The same analysis can be done for unknown powder and any crystal structure deposited in the Cambridge Structural Database (Groom et al., 2016). The great advantage of forensic requirements is that X-ray diffraction methods are non-destructive.

## Conclusions

The market for NPS is systematically growing. Successive addition of new compounds to the list of the substances being controlled all over the world does not discourage illegal laboratories from synthesizing novel deadly drugs. Therefore, there is a growing need to develop rapid and versatile analytical protocols as well as complementary techniques, which enable the provision of specific characterization for criminal investigations, thus preventing such illegal activity. In this article, we have presented a cooperative study toward the synthesis and characterization of selected NPS, with their intended application as CRMs for future use by law enforcement agencies.

We would like to highlight that the proposed non-routine analytical protocols for comprehensive characterization of NPS combining carefully selected physicochemical techniques, enables the generation of complementary information for selected NPS, namely those considered as emerging substances found on the illegal drug market.

We have successfully synthesized 7 NPS, those being known on the drug-market. An analytical procedure that combines chromatographic separation with the simultaneous identification of synthesized products using mass spectrometry with a Q/TOF type analyzer has been developed. The identity of the analyzed psychoactive substances was confirmed, and their purities have been determined.

Moreover, crystal structures of synthetic cannabinoids UR-144, NM-2201, 5F-PB-22 are presented in this article. A comparison between crystal and powder samples showed very good agreement. There are not many structures that we can qualify as new psychoactive substances among over one million deposited in CSD, so a lot must be done to facilitate the work of forensic scientists. X-ray diffraction methods, not commonly used by forensic scientists especially for synthetic cannabinoids, are non-destructive. This may be crucial for the evidence collected at the crime scene.

The completely characterized substances could serve as reference standards, which are intended to facilitate the work of law enforcement agencies. Thus, the combination of analytical instrumental techniques and X-ray diffraction may allow for rapid and credible studies of evidence collected at the crime scene. Moreover, such protocols enabling forensic scientists to gather supporting information may be used for judicial purposes as incontestable proof.

## Data Availability Statement

The datasets generated for this study can be found in the The Cambridge Crystallographic Data Center, CCDC 1842292-1842294, 1943878.

## Author Contributions

Syntheses of the compounds were performed by WK, PM and KG, the products were analyzed by electrospray Q/TOF-MS/MS, reversed-phase liquid chromatography (RP-HPLC) followed by detection with DAD detector. Q/TOF-MS and inductively coupled plasma with quadrupole mass spectrometer (ICP-QMS) by MM-K and EB, X-ray data collection and analyses were performed by MD and MC. RB was the holder of Grant No. DOB-BIO6/16/44/2014. The first draft of the manuscript was written by MD. All authors commented on previous versions of the manuscript, read, and approved the final manuscript, and contributed to the study conception and design.

## Conflict of Interest

The authors declare that the research was conducted in the absence of any commercial or financial relationships that could be construed as a potential conflict of interest.

## Footnotes

^1^European Monitoring Centre for Drugs and Drug Addiction. Available online at: http://www.emcdda.europa.eu/publications/edr/trends-developments/2019

^2^United Nations Office on Drugs and Crime. *Global SMART Programme*. Available online at: https://www.unodc.org/LSS/Page/NPS/GlobalSmart

^3^*United Nations Office on Drugs and Crime, Synthetic Cannabinoids in Herbal Products*. Available online at: http://www.unodc.org/documents/scientific/Synthetic_Cannabinoids.pdf

^4^*UNODC Laboratory and Scientific Section Portals*. Available online at: https://www.unodc.org/LSS/Announcement/Details/249b6412-8faf-46f1-8269-3dbd31d3bd40
