# Supplementary material for: Comprehensive Protocol for the Identification and Characterization of New Psychoactive Substances in the Service of Law Enforcement Agencies
Source: Front Chem. 2020 Sep 25;8:693. doi: 10.3389/fchem.2020.00693 (PMC7546804; doi:10.3389/fchem.2020.00693)
Supplement: Supplementary file 1 [file Data_Sheet_1.docx]

**Synthesis of compounds**


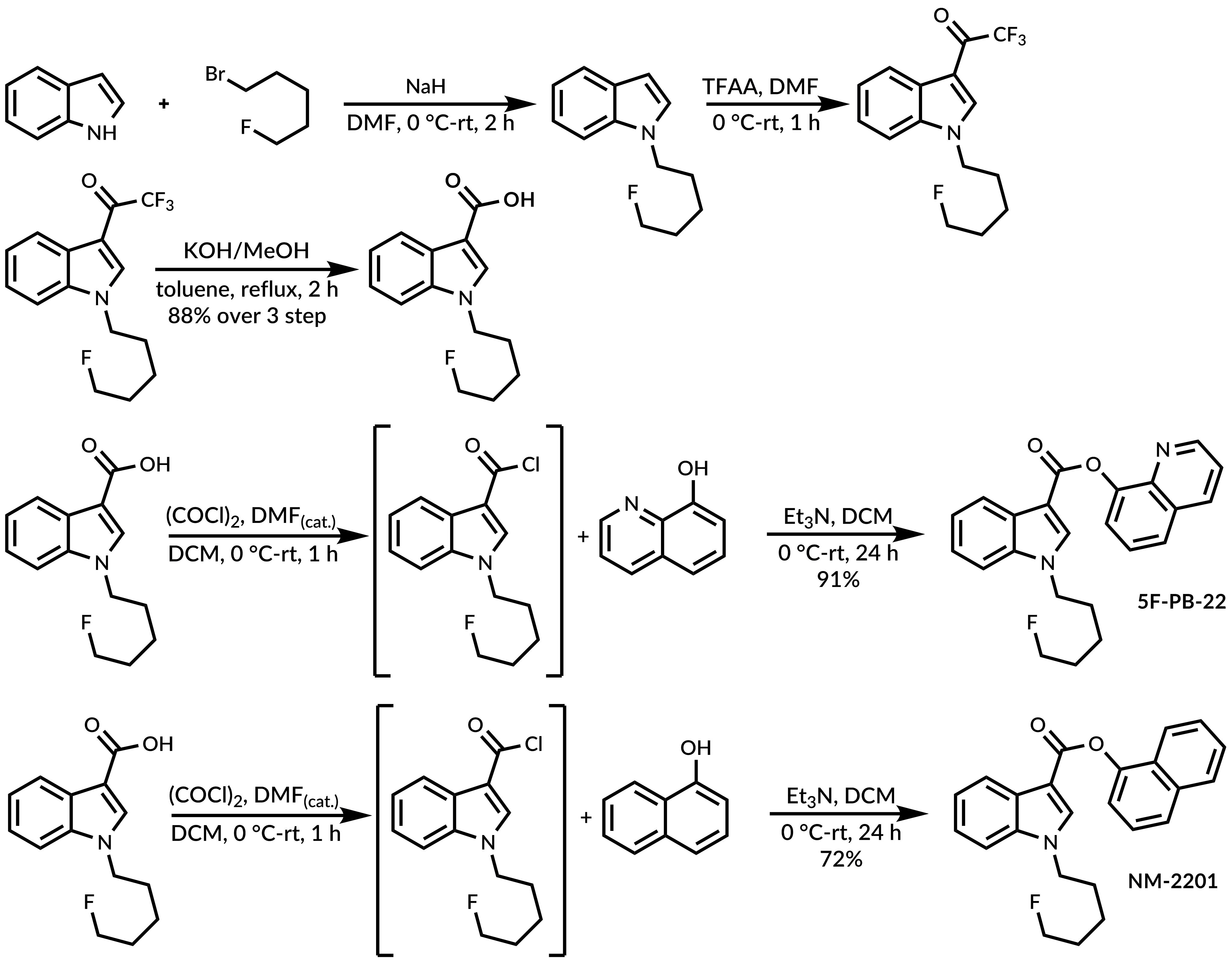


**Supplementary Scheme 1.** Synthesis of 5F-PB-22 and NM-2201.

***N-(5-fluoropentyl)-indole***

A solution of indole (2 g, 16.8 mmol, 1 equiv.) in dry DMF (40 mL) was cooled down to 0 °C and sodium hydride (60% dispersion in mineral oil, 0.81 g, 20.1 mmol, 1.2 equiv.) was added portion-wise during 10 min. A resulted suspension was stirred over 30 min allowing to reach room temperature. After being cooled down to 0 °C again, 5-fluoropentyl bromide (3.12 g, 18.5 mmol, 1.1 equiv.) was added dropwise. The reaction mixture was stirred at RT until substrate’s disappearance acc. to TLC (10% ethyl acetate in hexane), usually 2 h. This reaction mixture was directly use in next step.

***N-(5-fluoropentyl)-3-(trifluoroacetyl)-indole***

Reaction mixture was cooled down to 0 °C and trifluoroacetic acid anhydride, TFAA (5.2 mL, 36,9 mmol, 2.2 equiv.) was added dropwise during 15 min. Intensively red solution was stirred for 1 h allowing to reach RT. After substrate disappearance (acc. to TLC), crushed ice (150 g) was added and the pink precipitate was filtered, which was used in the next step without purification.

***N-(5-fluoropentyl)-indole-3-carboxylic acid***

A solution of N-(5-fluoropentyl)-3-(trifluoroacetyl)indole in toluene (6 mL) was added dropwise to the boiling mixture of KOH (3.10 g, 55.4 mmol, 3.3 equiv.) in methanol (15 mL). A reaction mixture was stirred for 2 h in boiling methanol and then was cooled down to RT. Water (50 mL) was added and the layers were separated. Water layer was acidified with concentrated hydrochloric acid to pH=1 and extracted with DCM twice. Organic fractions were dried under MgSO_4_, filtered and evaporated. Crude product was dissolved in EtOAc and precipitated with pentane. The desired product was obtained as a colorless solid in 88% overall yield.

***NM-2201***

A solution of N-(5-fluoropentyl)-indole-3-carboxylic acid (0.64 g, 2.54 mmol, 1 equiv.) and one drop of DMF in dry dichloromethane (6 mL) was cooled down to 0 °C. Oxalyl chloride (0.44 mL, 5.08 mmol, 2 equiv.) was added dropwise and the reaction mixture was stirred for 1 h allowing to reach RT. A mixture was evaporated to dryness and remaining solid was dissolved in a mixture of DCM (14 mL) and triethylamine (1,25 mL, 8.89 mmol, 3.5 equiv.). A solution was cooled down to 0 °C and 1-naphthol (0.44 g, 3.04 mmol, 1.2 equiv.) in DCM (14 mL) was added dropwise. Cooling bath was removed and resulted suspension was stirred for 20 h. The mixture was evaporated to dryness, dissolved in EtOAc, and extracted with saturated solution of NaHCO_3_ and brine. Organic fractions were dried over MgSO_4_ and filtered through short pad of silica gel. Crystallization from mixture of EtOAc, Et_2_O, and pentane allow to obtain pure product in 72 % yield as colorless solid.

***5F-PB-22***

A solution of N-(5-fluoropentyl)-indole-3-carboxylic acid (0.64 g, 2.54 mmol, 1 equiv.) and one drop of DMF in dry dichloromethane (6 mL) was cooled down to 0 °C. Oxalyl chloride (0.44 mL, 5.08 mmol, 2 equiv.) was added dropwise and the reaction mixture was stirred for 1 h allowing to reach RT. A mixture was evaporated to dryness and remaining solid was dissolved in a mixture of DCM (14 mL) and triethylamine (1,25 mL, 8.89 mmol, 3.5 equiv.). A solution was cooled down to 0 °C and 8-hydroxyquinoline (0.44 g, 3.04 mmol, 1.2 equiv.) in DCM (14 mL) was added dropwise. Cooling bath was removed and resulted suspension was stirred for 20 h. The mixture was evaporated to dryness, dissolved in EtOAc, and extracted with saturated solution of NaHCO_3_ and brine. Organic fractions were dried over MgSO_4_ and filtered through short pad of silica gel. Crystallization from mixture of EtOAc and hexane allow to obtain pure product as a colorless solid in 91% yield.


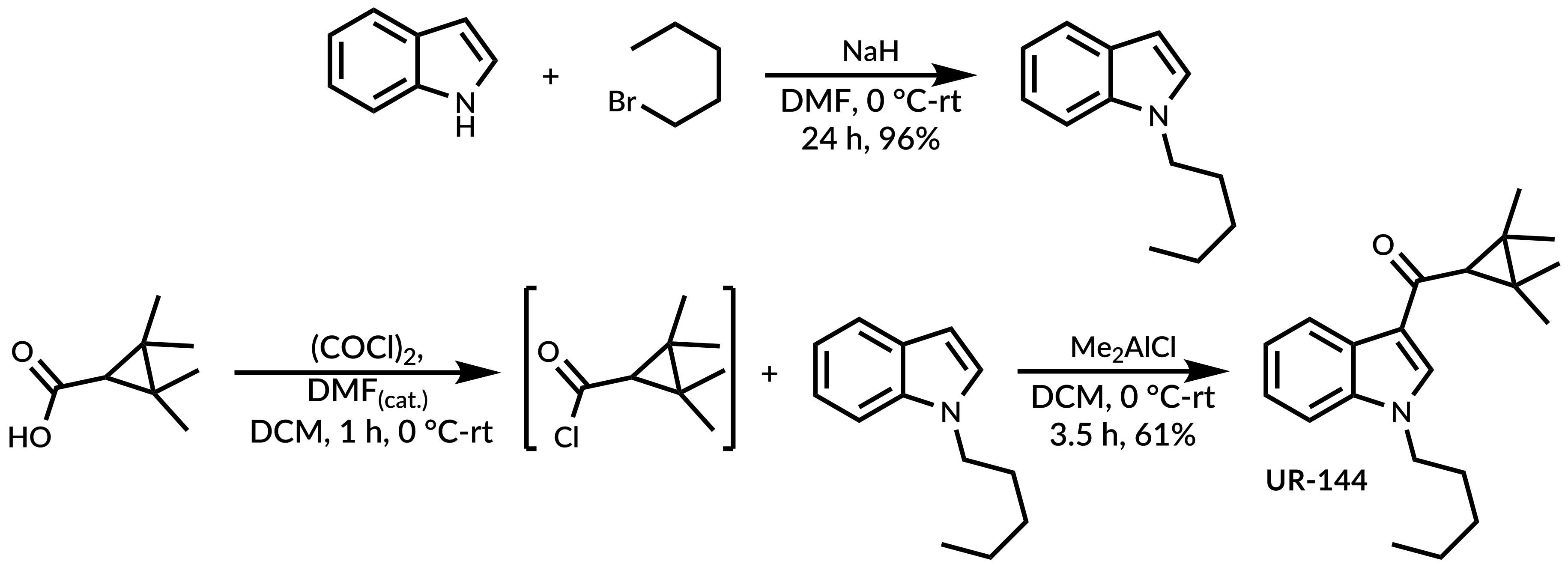


**Supplementary Scheme 2.** Synthesis of UR-144.

***N-pentylindole***

A solution of indole (6.44 g, 54 mmol, 1 equiv.) in dry DMF (100 mL) was cooled down to 0 °C and sodium hydride (60% dispersion in mineral oil, 2.60 g, 64.8 mmol, 1.2 equiv.) was added portion-wise during 10 min. A suspension was stirred over 30 min allowing to reach room temperature. After being cooled down to 0 °C again, pentyl bromide (7.4 mL, 59.4 mmol, 1.1 equiv.) was added dropwise. The reaction mixture was stirred at RT until substrate disappearance acc. to TLC (10% ethyl acetate in hexane), usually 2 h. Water (10 mL) was added and solvents were evaporated. Residue was dissolved in methyl-*tert*-butyl ether and washed with water and brine. Organic layer was dried over MgSO_4_, filtered and evaporated. Crude product was purified on silica gel flash chromatography (2% - 10% ethyl acetate in hexane). The desired product was obtained in 96% yield as yellowish oil.

***UR-144***

A solution of 2,2,3,3-tetramethylcyclopropanecarboxylic acid (1.00 g, 7 mmol, 1 equiv.) and one drop of DMF in DCM (36 mL) was cooled down to 0 °C. Oxalyl chloride (1.20 mL, 14 mmol, 2 equiv.) was added dropwise and the reaction mixture was stirred for 1 h allowing to reach RT. A mixture was evaporated to dryness on membrane pump and remaining solid was dissolved in DCM (14 mL).

In separated flask solution of N-pentylindole in dry DCM (14 mL) was prepared and cooled down to 0 °C. 1M solution of Me_2_AlCl (8.1 mL, 8.1 mmol, 1.5 equiv.) in hexane was added. Reaction mixture was stirred for 30 min and previously prepared solution of acid chloride was added. Cooling bath was removed and reaction mixture was stirred for another 3 h. A reaction mixture was cooled down to 0 °C and 1M solution of hydrochloric acid (30 mL) was added. Organic layer was separated, washed with solution of NaHCO_3_ and brine, dried over MgSO_4_, filtered and evaporated. Crude product was purified on silica gel flash chromatography and crystallized from isopropanol/pentane mixture. Pure product as colorless crystals was obtain in 61% yield.


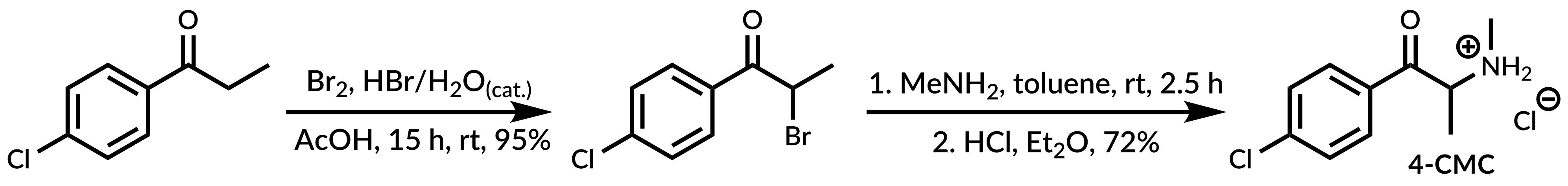


**Supplementary Scheme 3.** Synthesis of 4-CMC.

***2-Bromo-1-(4-chlorophenyl)propan-1-one***

A solution of Br_2_ (2.25 mL, 43 mmol, 1.05 equiv.) in glacial acetic acid (5 mL) was added dropwise during 1 h to the solution of 4-chloropropiophenone (6.9 g, 41 mmol, 1 equiv.) in glacial acetic acid (40 mL). A reaction mixture was stirred at RT for 24 h. Solvent was evaporated and crude product was purified by crystallization (ethyl acetate/ pentane). The desired product was obtained in 88% yield as a colorless crystals.

***4-CMC***

A solution of 2-bromo-1-(4-chlorophenyl)propan-1-one (990 mg, 4 mmol, 1vequiv.) in toluene (5 mL) was added dropwise during 1 h (syringe pump) to 33% methylamine solution in ethanol (1.1 mL, 8 mmol, 2 equiv.). A reaction mixture was stirred at RT for 2 h. 1M NaOH (4 mL) water solution was added followed by Et_2_O (5 mL). Organic layer was separated, dried over Na_2_SO_4_, and filtered. 1M HCl solution in Et_2_O (4.2 mL) was added to the cold solution. Precipitate was filtered and washed with Et_2_O. The desired product was obtained in 70% yield as a colorless powder.


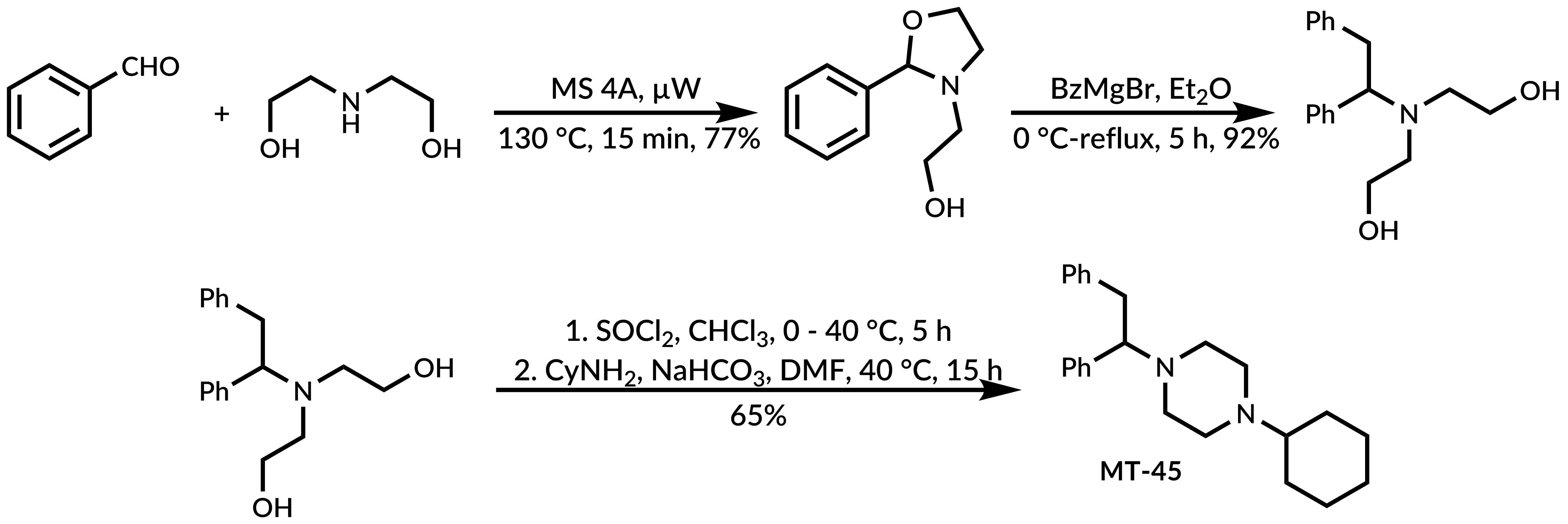


**Supplementary Scheme 4** Synthesis of MT-45.

***N-(2-hydroxyethyl)-2-phenyloxazolidine***

Benzaldehyde (2 g, 18.8 mmol, 1.25 equiv.), diethanolamine (1.50 g, 14.5 mmol, 1 equiv.) and finely powdered 4A molecular sieves (1 g) were heated in microwave reactor (150 W) for 15 min. A reaction mixture was filtered through a celite pad and washed with DCM. Solvent was evaporated. Unreacted starting materials was evaporated on Kugelrohr apparatus under high vacuum. The desired product was obtained as a yellowish oil and was used without further purification.

***N,N-bis(2-hydroxyethyl)-1,2-diphenylethylamine***

A solution of previously prepared oxazolidine (550 mg, 2.85 mmol, 1 equiv.) in Et_2_O (10 mL) was slowly added to freshly prepared solution of benzylmagnesium bromide in Et_2_O (0.3 M, 30mL, 3 equiv.) at 0 °C. A reaction mixture was stirred at 35 °C for 5 h. Brine (15 mL) was added and organic layer was separated. Organic layer was dried over Na_2_SO_4_, filtered and evaporated. Crude product was purified using flash chromatography (DCM/methanol 0 to 20%). The desired product was obtained as a yellowish oil in 92% yield.

**MT-45**

Thionyl chloride (0.4 mL, 5.5 mmol, 3 equiv.) was slowly added to cold solution of *N*,*N*-bis(2-hydroxyethyl)-1,2-diphenylethylamine (520  mg, 1.8 mmol, 1 equiv.) in chloroform (10 mL). A reaction mixture was stirred at 40 °C for 5 h. Solvent was evaporated, residue was dissolved in 10% mixture of ethyl acetate in hexane and filtered through pad of neutral aluminum oxide. Solvents were evaporated and residue was redissolved in dry DMF (4mL). NaHCO_3_ (470 mg, 5.6 mmol, 4 equiv) and cyclohexylamine (320 μl, 2,8 mmol, 2 equiv.) was added. Reaction mixture was stirred at 100 °C for 20 h. Solvent was evaporated and residue was dried on high vacuum to remove remaining cyclohexylamine. MTBE (20 mL) and 10% LiCl solution (10 mL) were added. Layers were separated. Organic phase was dried over Na_2_SO_4_, filtered, and evaporated. Crude product was purified using flash chromatography (on neutral aluminum oxide) and crystallized from cold pentane. The desired product was obtained as a beige crystals in 65% yield.


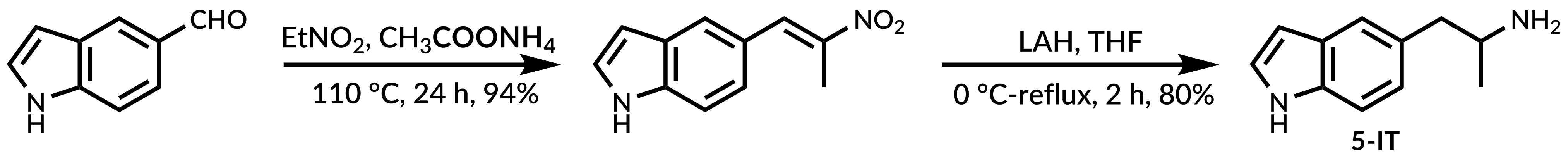


**Supplementary Scheme 5** Synthesis of 5-IT.

**5-(2-nitroprop-1-en-1-yl)indole**

Ammonium acetate (0.69 g, 9 mmol, 1 equiv.) was added to a solution of indole-5-carbaldehyde (2.67 g, 18 mmol, 2 equiv.) in nitroethane (18 mL). A reaction mixture was stirred at 110 °C for 24 h. A solution was cooled down to RT and water (50 mL) was added. Precipitate was filtered and dried. Product was obtained in 94% yield as orange powder.

**5-IT**

A solution of 5-(2-nitropropenylo)indole (500 mg, 2.5 mmol, 1 equiv.) in dry THF (5 mL) was slowly added to a suspension of LAH (560 mg, 14.8 mmol, 6 equiv.) in 25 mL of dry THF. A reaction mixture was stirred in boiling THF for 2 h. 15% NaOH solution (1 mL) and water (2 mL) was added. Precipitate was filtered through pad of celite. Filtrate was evaporated and the residue was purified using silica gel flash chromatography (DCM/methanol 10%). The desired product was obtained in 80% yield as a beige powder.


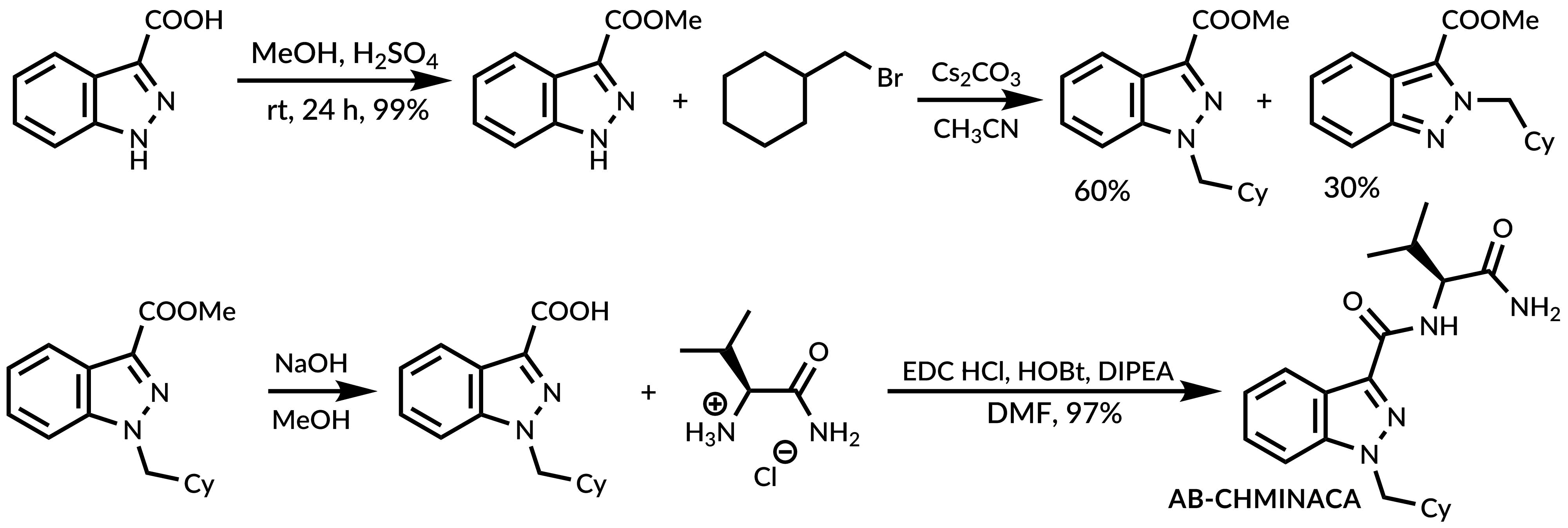


**Supplementary Scheme 6** Synthesis of AB-CHMINACA.

***Methyl 1H-indazole-3-carboxylate***

Concentrated sulfuric acid (4 mL) was added to the solution of indazole-3-carboxylic acid (2.0 g, 12.3 mmol) in methanol (40 mL). A reaction mixture was stirred at RT for 16 h. Solvent was evaporated, residue was redissolved in DCM, and washed with water. Organic phase was dried over MgSO_4_, filtered, and evaporated. The desired product was obtained in 99% yield as a colorless powder and used without further purification.

**Methyl 1-(cyclohexylmethyl)-indazole-3-carboxylate**

Cs_2_CO_3_ (3.55 g, 10.8 mmol, 2 equiv.) was added to the solution of methyl 1H‑indazole-3-carboxylate (0.88 g, 5.4 mmol, 1 equiv.) in dry CH_3_CN (15 mL). A suspension was stirred at RT for 30 min and (bromomethyl)cyclohexane (2.1 g, 11.9 mmol, 2.2 equiv.) was added. A reaction mixture was stirred at RT for 20 h. Precipitate was filtered and solution was washed with brine. Organic phase was dried over MgSO_4_, filtered and evaporated. Pure product was obtained in 60% yield after flash chromatography (ethyl acetate/hexane 25-50%).

**1-(cyclohexylmethyl)-indazole-3-carboxylic acid**

NaOH (330 mg, 8.3 mmol, 2.5 equiv.) was added to the solution of methyl 1‑(cyclohexylmethyl)-indazole-3-carboxylate (3.30 mmol) in methanol (34 mL). A reaction mixture was stirred at RT for 24 h and then acidified to pH=5. Methanol was evaporated and residue was redissolved in DCM. Organic layer was washed with water and brine. Crude product, after drying and evaporation, was used without further purification

**AB-CHMINACA** and 2-isomer AB-CHMINACA

EDC·HCl (0.78 g, 4.08 mmol, 1.35 eq.), HOBt·H_2_O (0,65 g, 4.23 mmol, 1.4 equiv.) and DIPEA (2 mL, 12.1 mmol, 4 equiv.) was added to the solution of 1-(cyclohexylmethyl)-indazole-3-carboxylic acid (0.78g, 3.02 mmol, 1 equiv.) and *L*-vanilamide hydrochloride (0,62 g, 4.08 mmol, 1.35 eq.) in dry DMF (15 mL). Reaction mixture was stirred at RT for 24 h. Solvent was evaporated and the residue was dissolved in DCM. Organic phase was washed with water and brine. Crude product after drying, filtration, and evaporation was purified using flash chromatography (10%-50% EtOAc in hexane). The desired product was obtained in 97% yield.

The same procedure was used to obtain the 2-isomer AB-CHMINACA giving the product in 82% yield after two steps.

**X-ray diffraction**

**Supplementary Table 1.** Crystal data and structure refinement for 4-CMC.

| Compound | **4-CMC** |
| --- | --- |
| Empirical formula | C_10_H_13_N_1_O_2_Cl_2_ |
| Formula weight | 250.11 |
| Temperature [K] | 130 |
| Space group | *P2_1_/n* |
| Unit cell dimensions |  |
| *a* [Å] | 6.0503(2) |
| *b* [Å] | 8.0453(2) |
| *c* [Å] | 24.1827(7) |
| α [°] | 90 |
| β [°] | 90.4190(10) |
| γ [°] | 90 |
| Volume *V* [Å^3^] | 1177.10(6) |
| Z [molecules/cell] | 4 |
| *D*_calculated_ [g cm^-3^] | 1.411 |
| Absorption coefficient  μ/ mm^-1^ | 0.531 |
| *θ* range for data collection [°] | 2.668-26.000 |
| Limiting indices | -7 < = *h* = > 7 |
|  | -9 < = *k* = > 9 |
|  | -29 < = *l* = > 29 |
| Reflections collected/unique | 12777/2316 |
| Data/parameters | 2316/146 |
| Goodness of Fit | 1.090 |
| Final *R* index (*I* > 2σ) | 0.0256 |
| w*R*^2^ | 0.0620 |
| Largest diff. Peak and hole [Å^-3^] | 0.30 and -0.20 |
| CCDC | 1943878 |


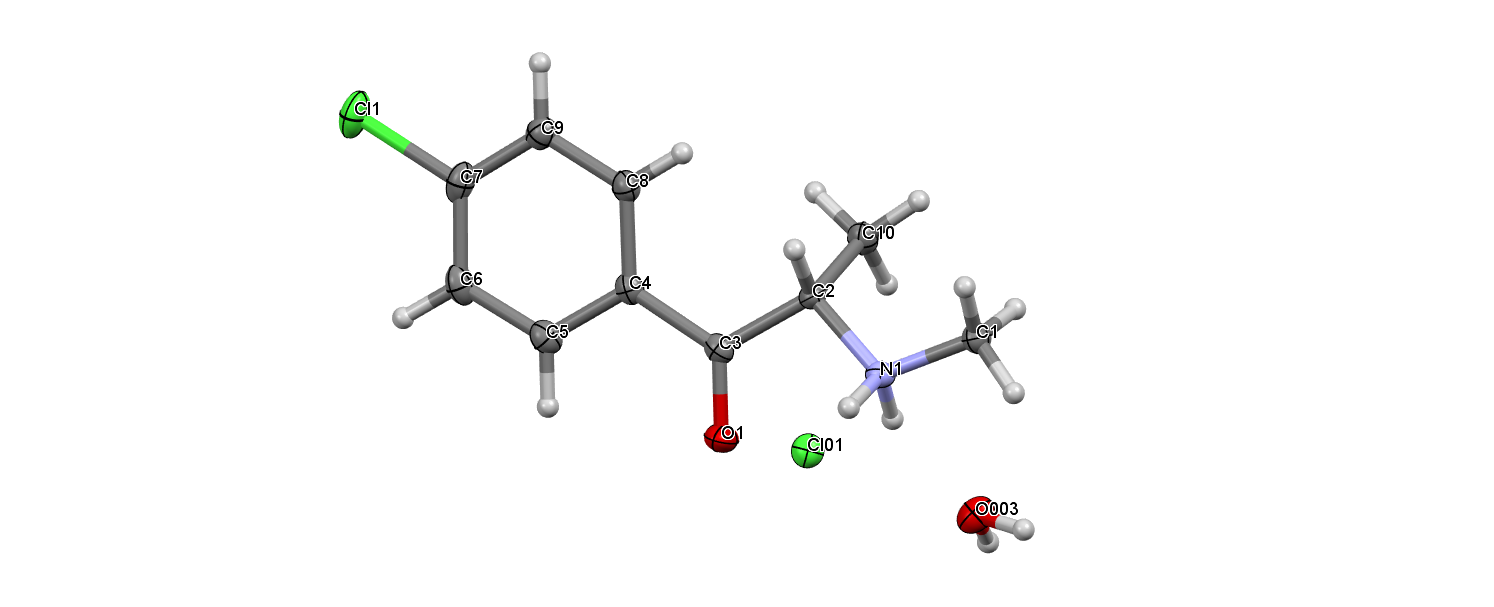


**Supplementary Figure 1.** The molecular structure of 4-CMC showing atom numbering and displacement ellipsoids at the 50% probability level.


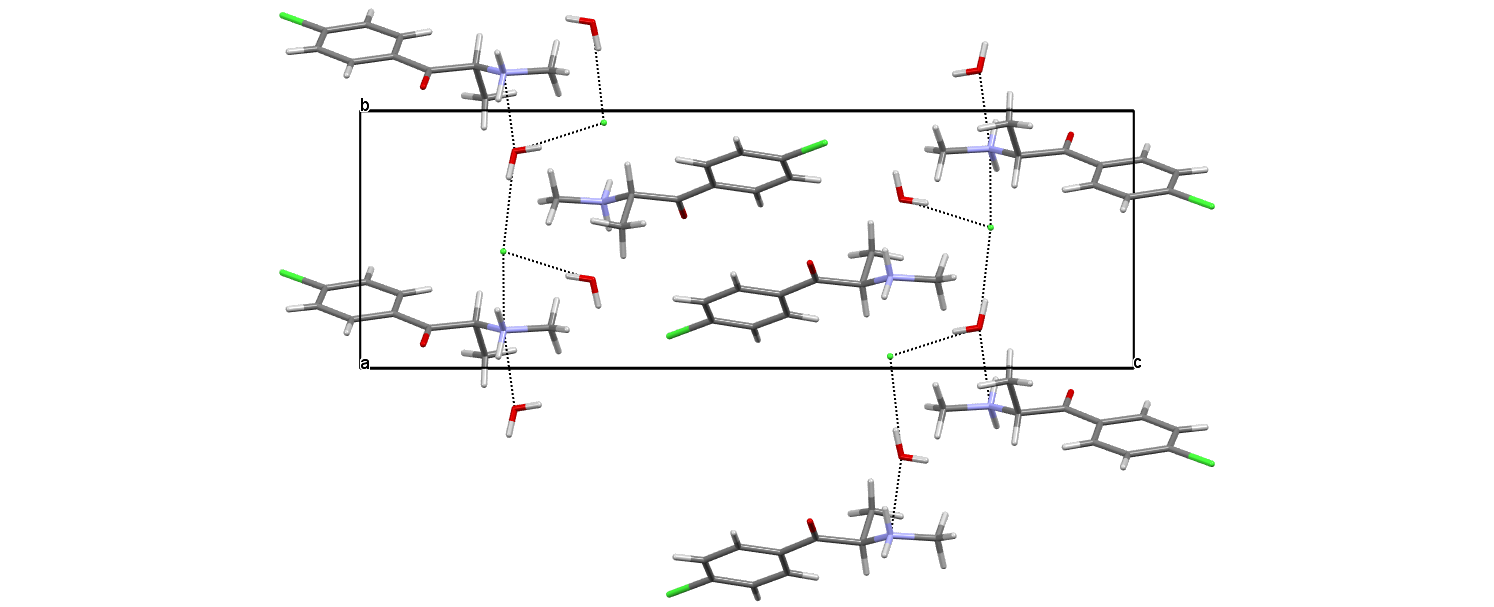


**Supplementary Figure 2.** Crystal packing and Short interactions in the crystal structure of 4-CMC.

**^1^H NMR spectra**


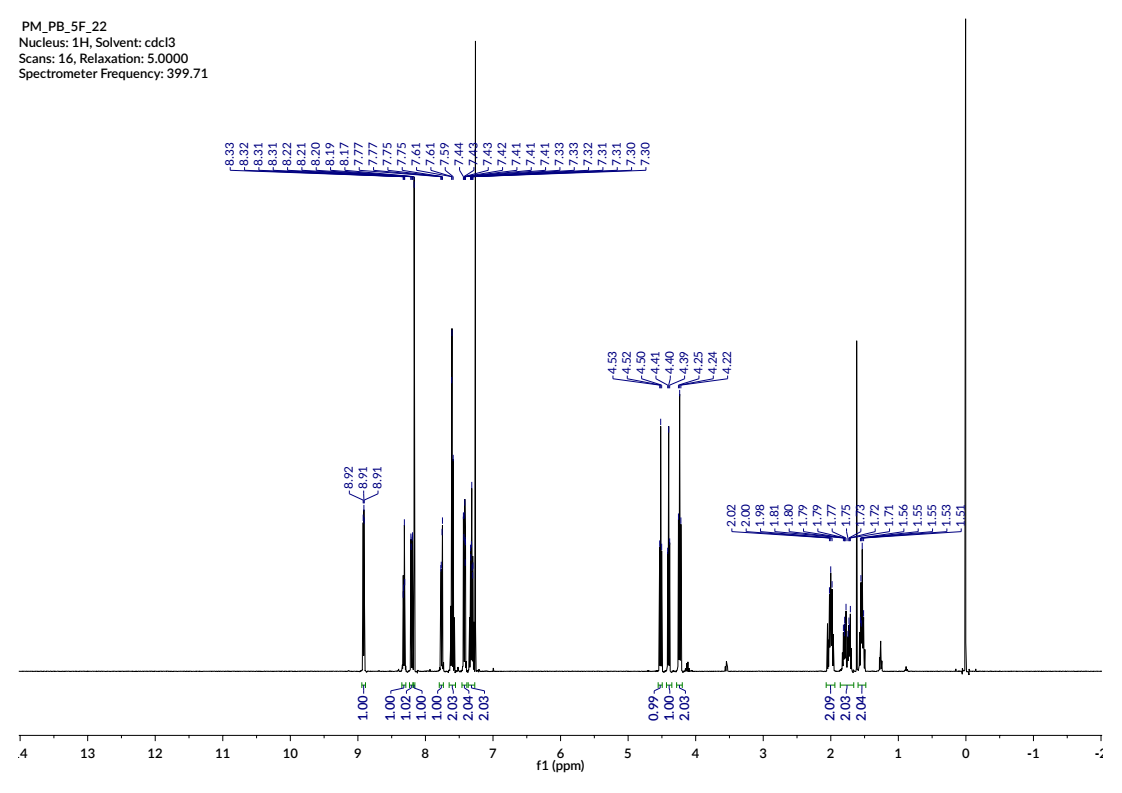


**Supplementary Figure 3.** ^1^H NMR spectrum of 5F-PB-22.


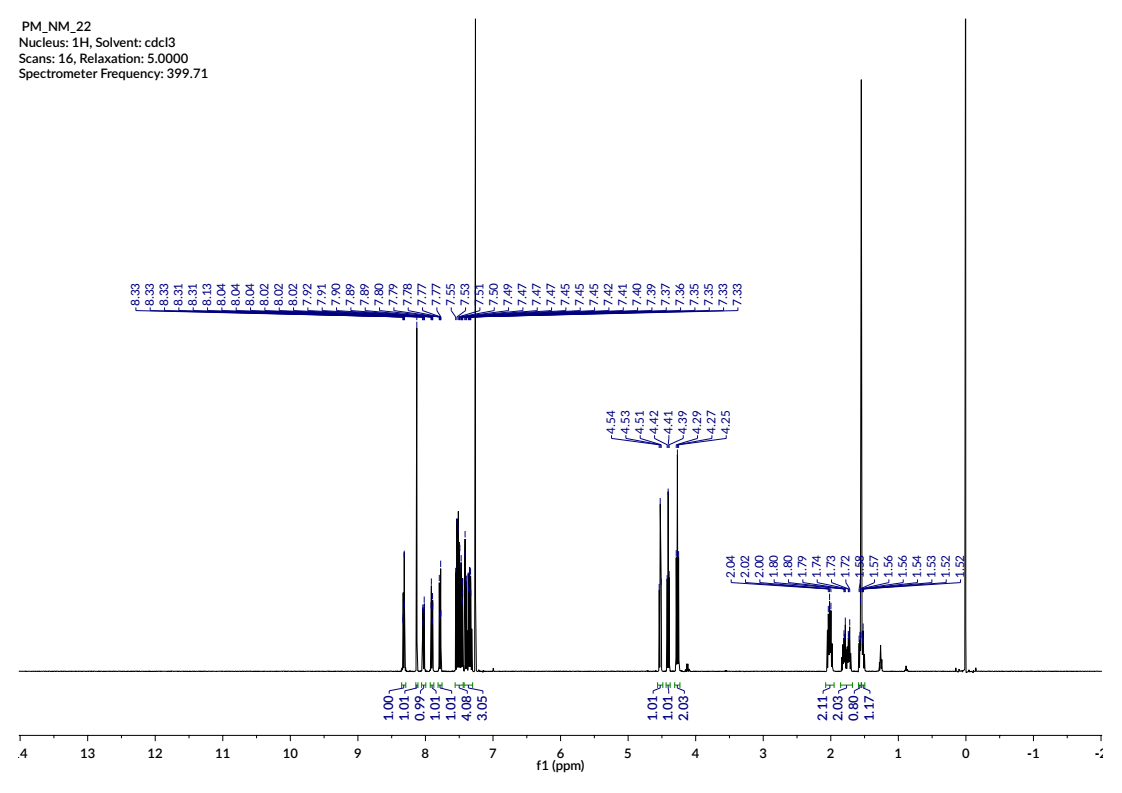


**Supplementary Figure 4.** ^1^H NMR spectrum of NM-2201.


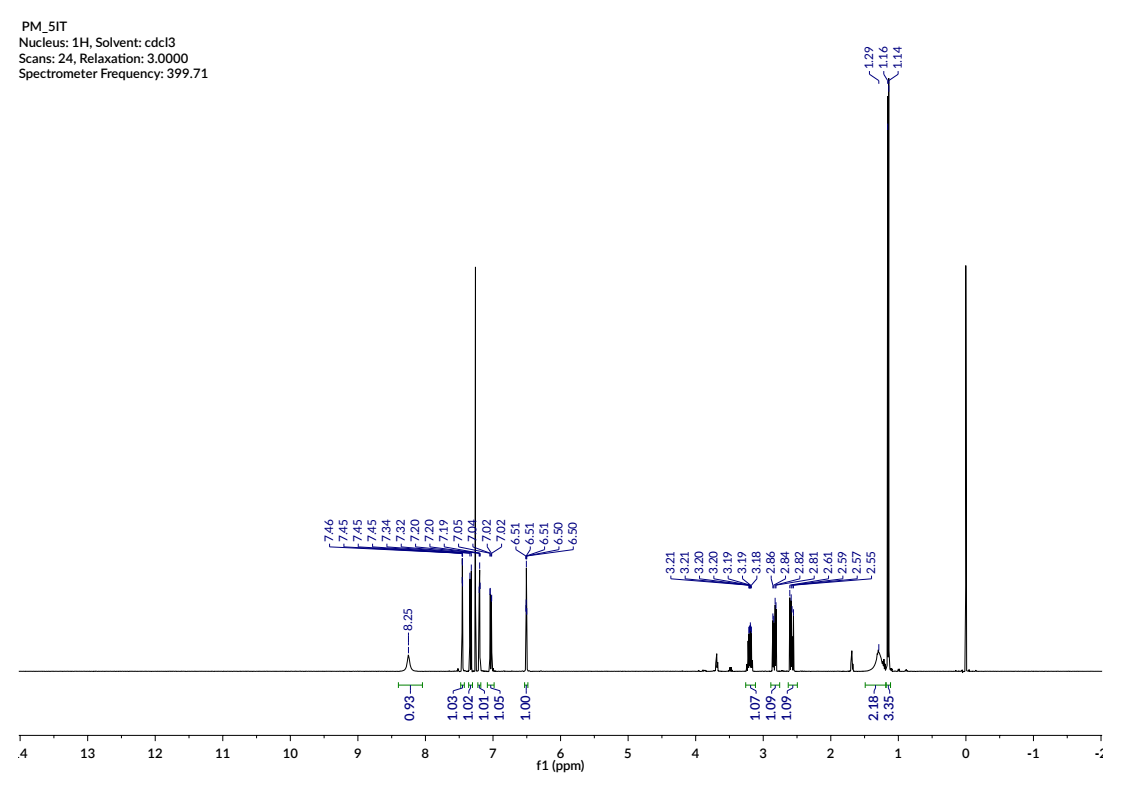


**Supplementary Figure 5**. ^1^H NMR spectrum of 5-IT.


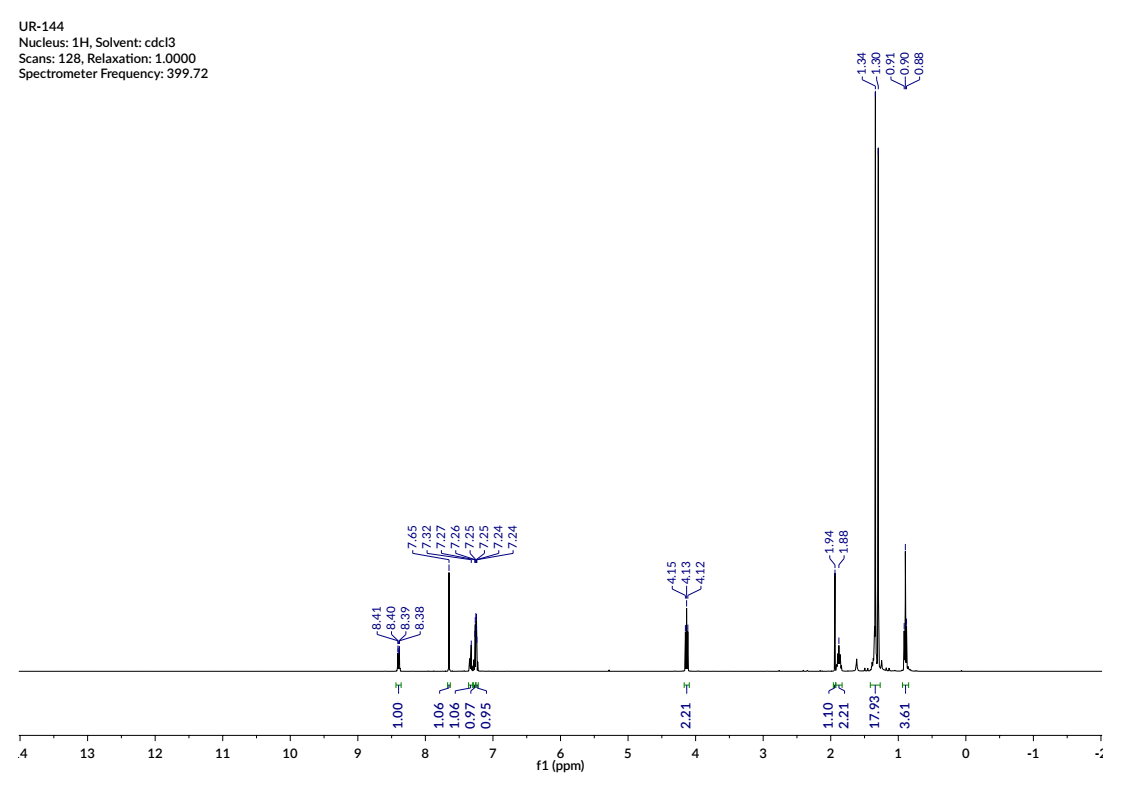


**Supplementary Figure 6.** ^1^H NMR spectrum of UR-144.


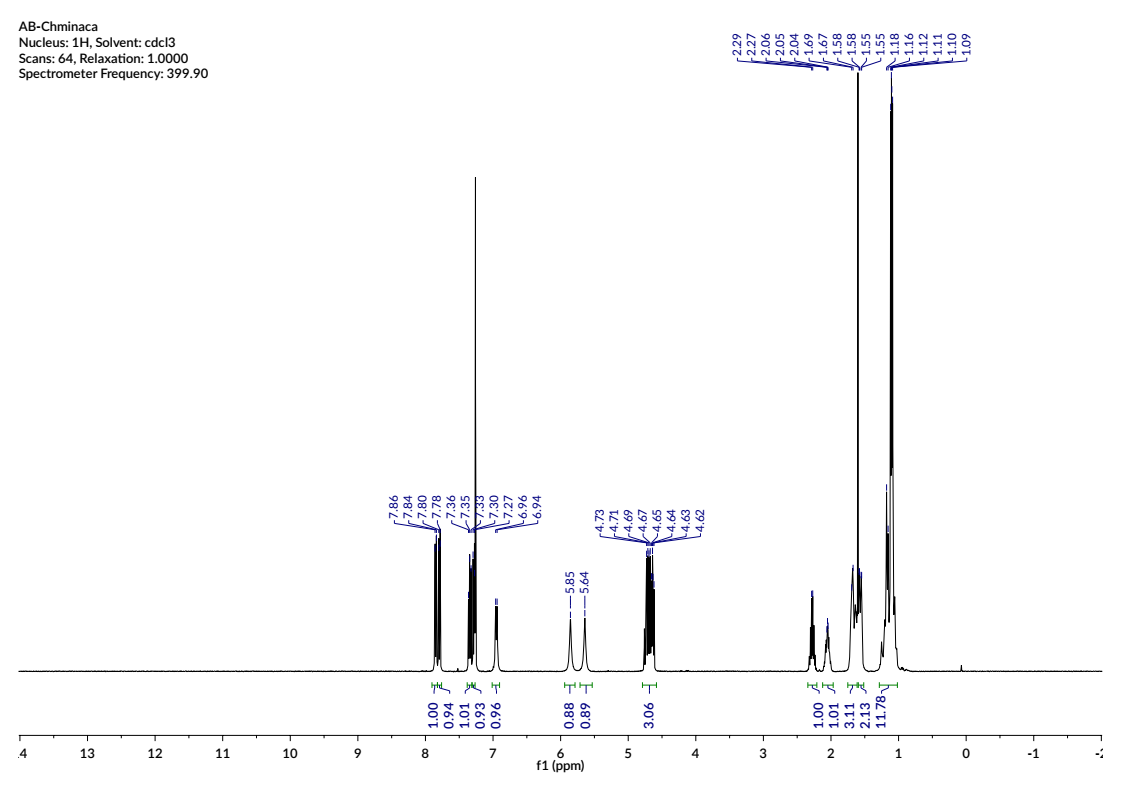


**Supplementary Figure 7.** ^1^H NMR spectrum of AB-CHMINACA.


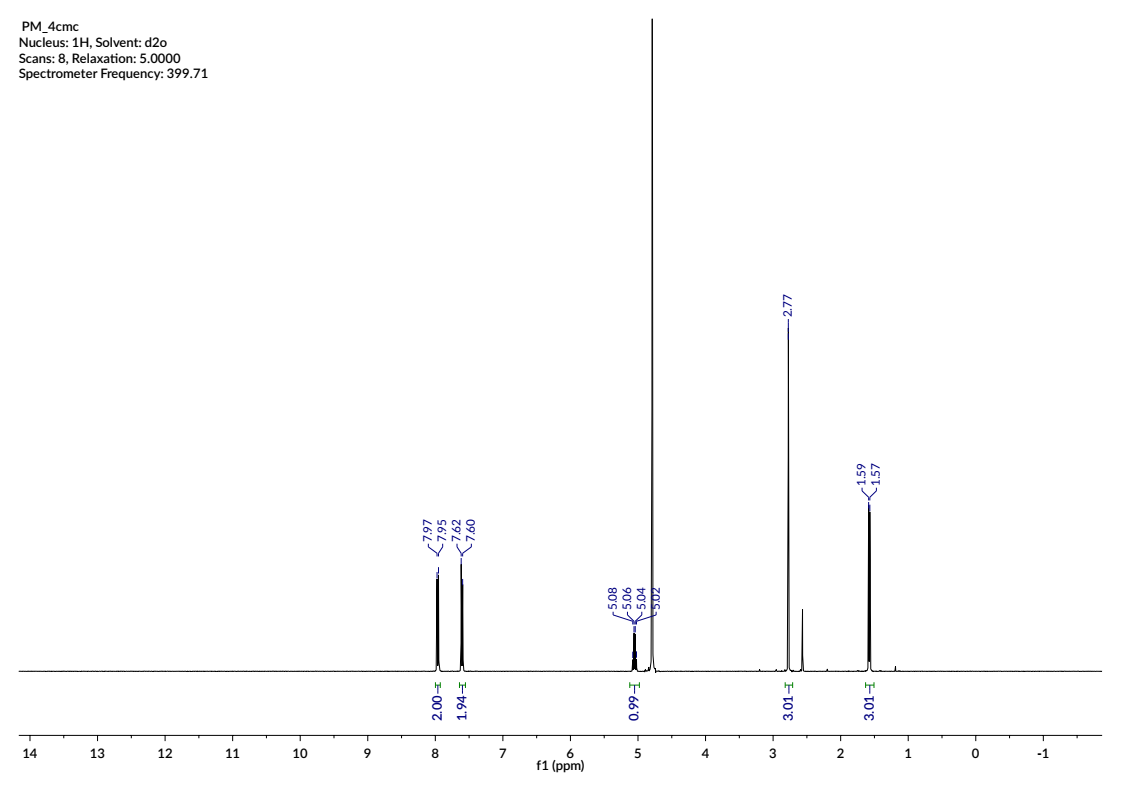


**Supplementary Figure 8.** ^1^H NMR spectrum of 4-CMC.


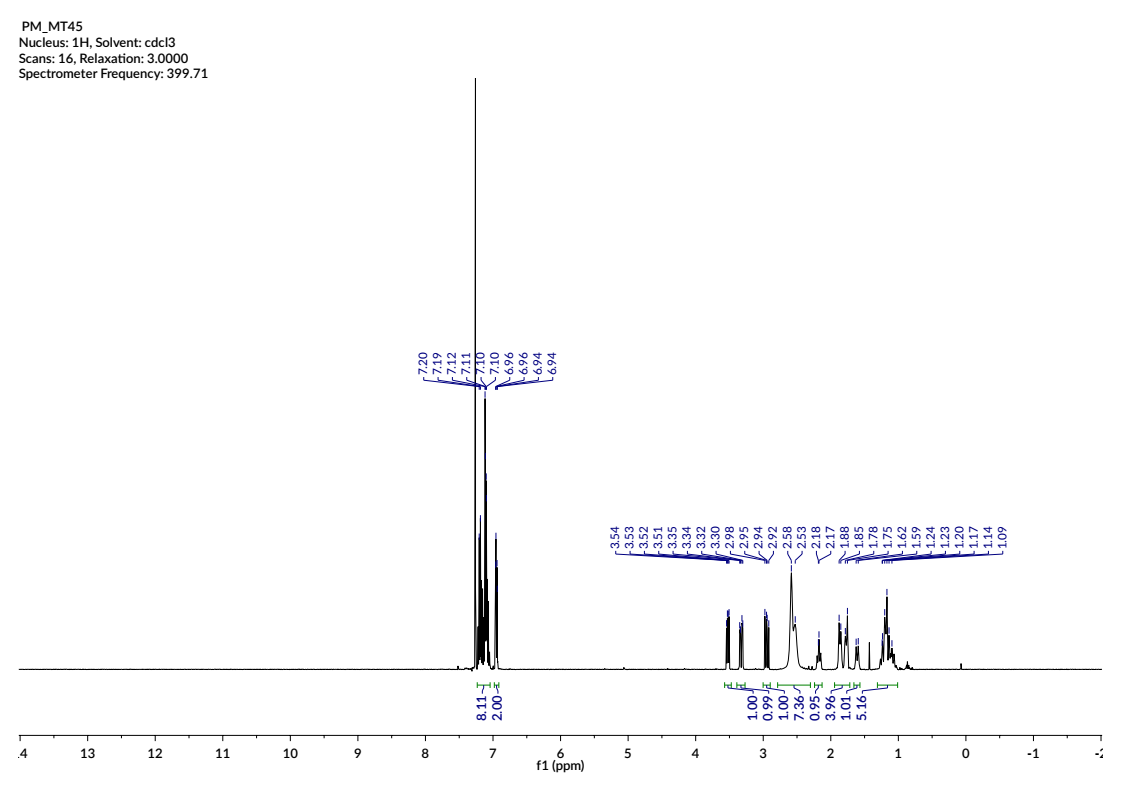


**Supplementary Figure 9.** ^1^H NMR spectrum of MT-45.


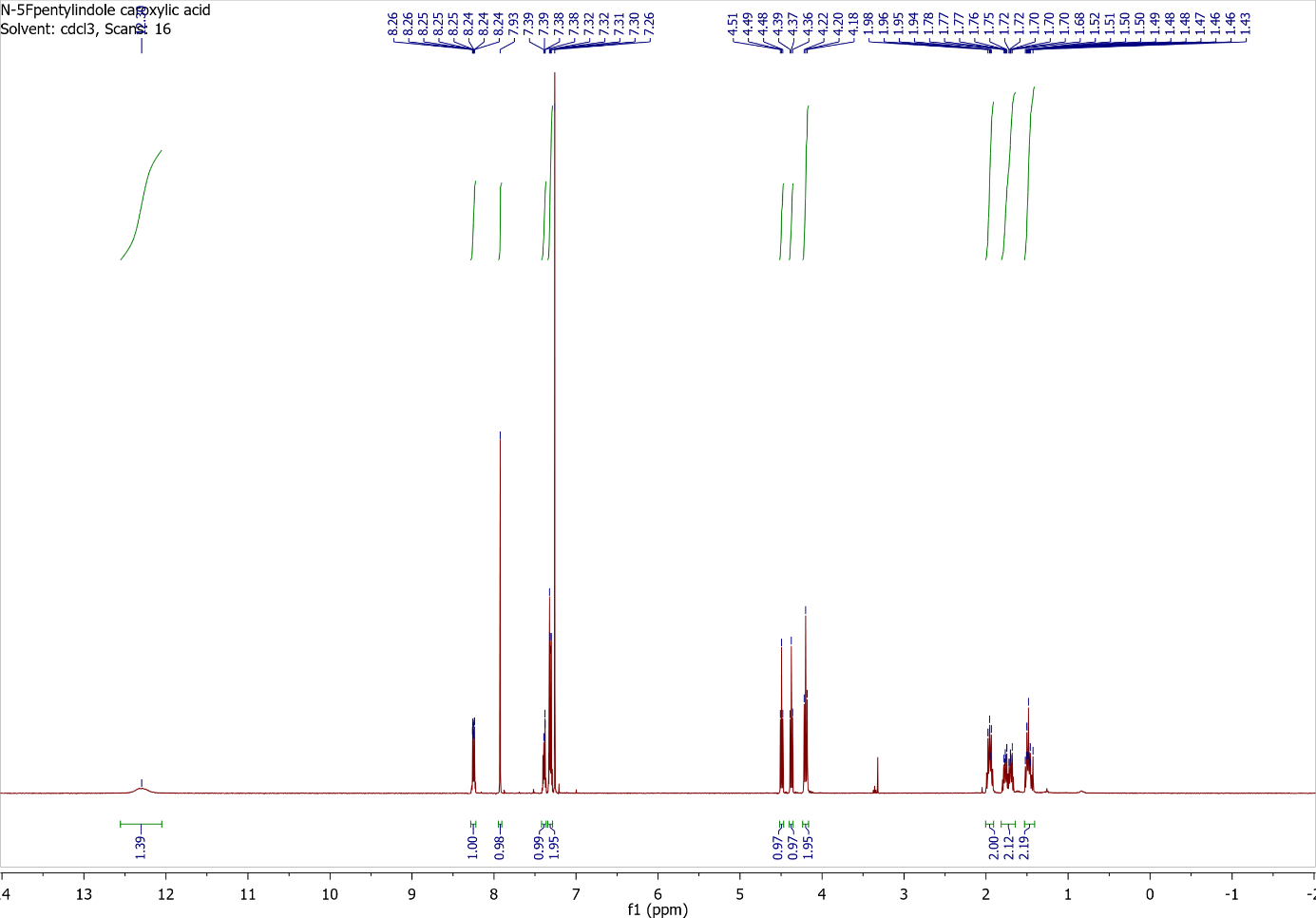


**Supplementary Figure 10.** ^1^H NMR spectrum of **N-(5-fluoropentyl)-indole-3-carboxylic acid.**


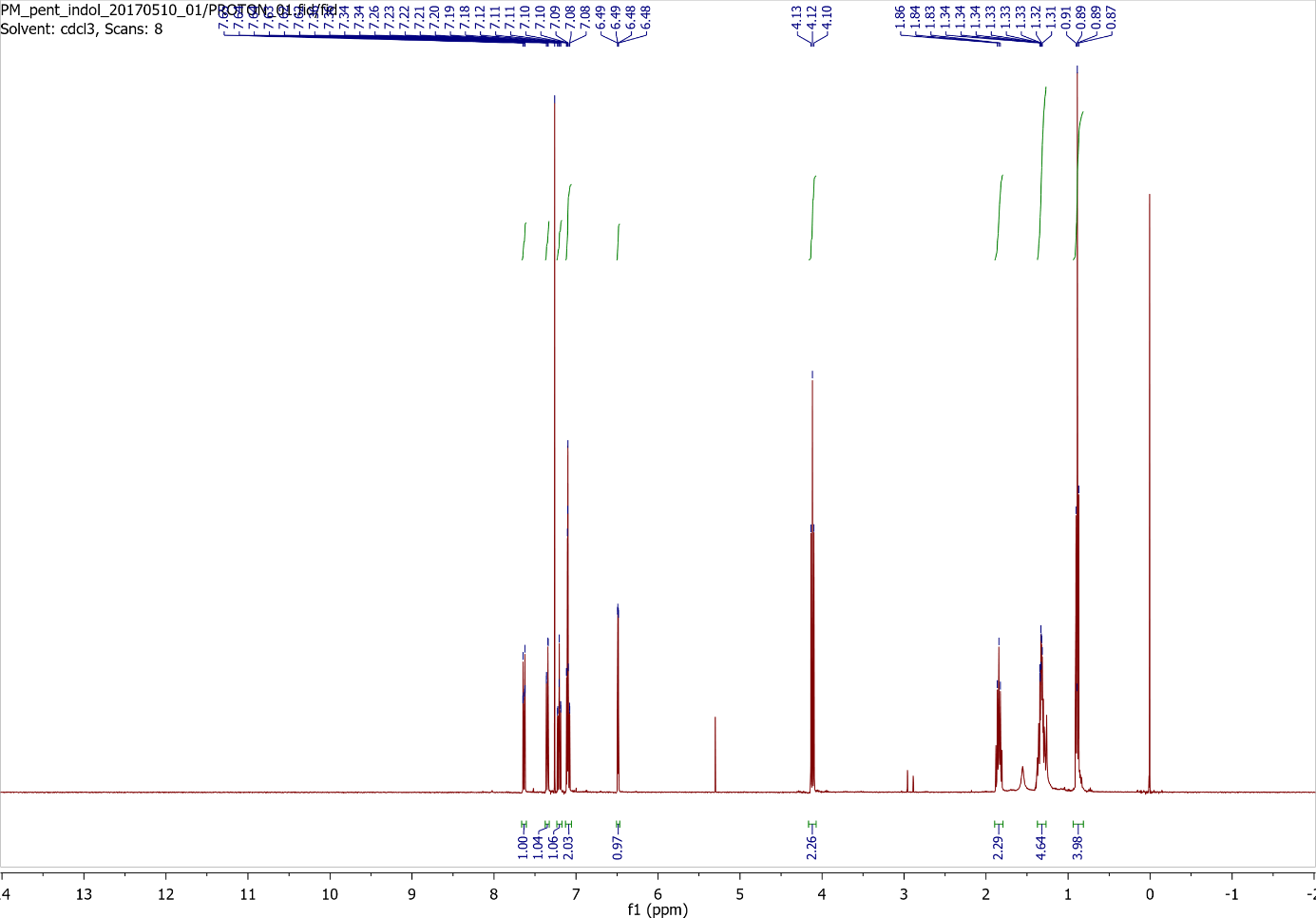


**Supplementary Figure 11.** ^1^H NMR spectrum of ***N*-pentylindole.**


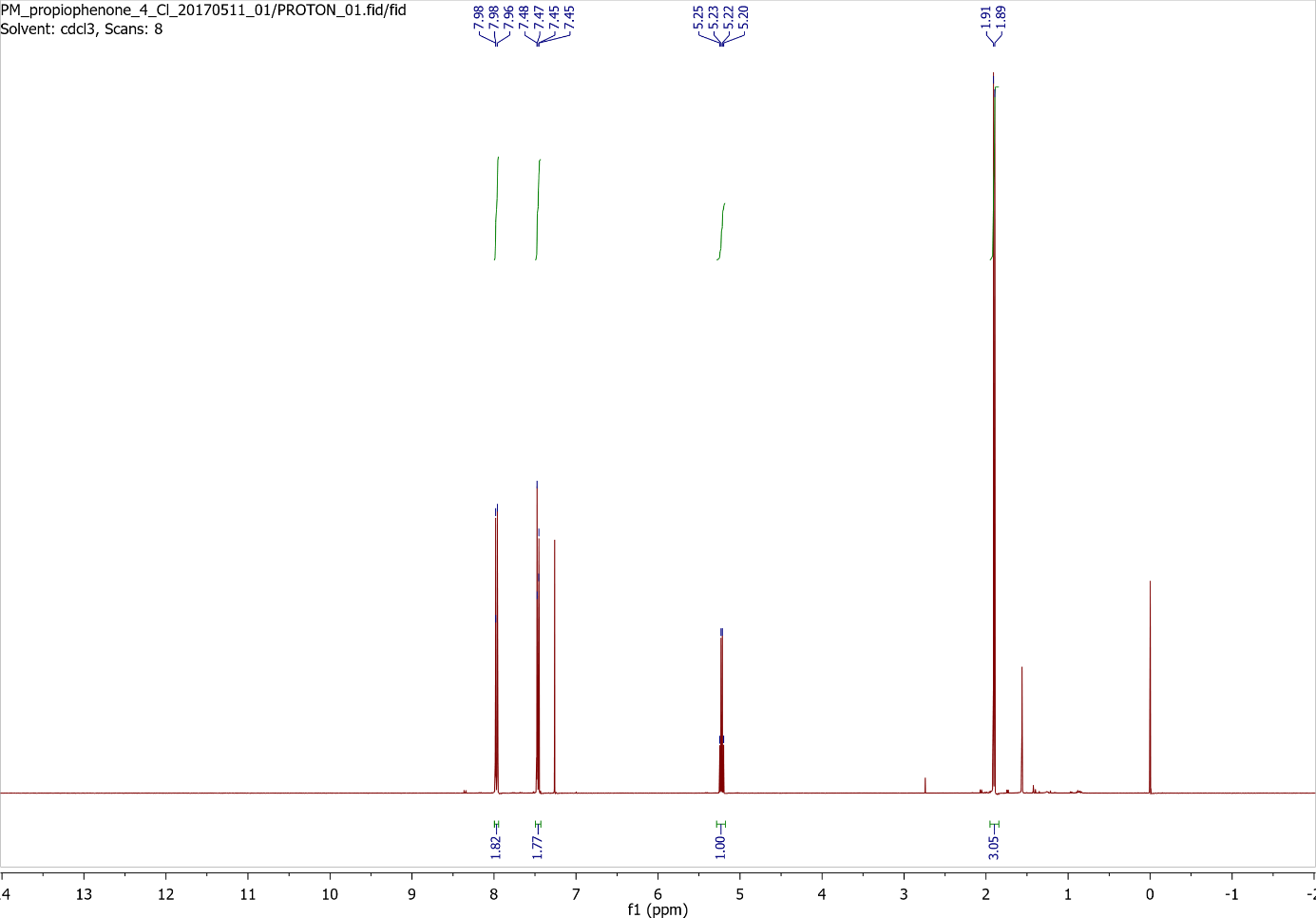


**Supplementary Figure 12.** ^1^H NMR spectrum of **2-Bromo-1-(4-chlorophenyl)propan-1-one.**


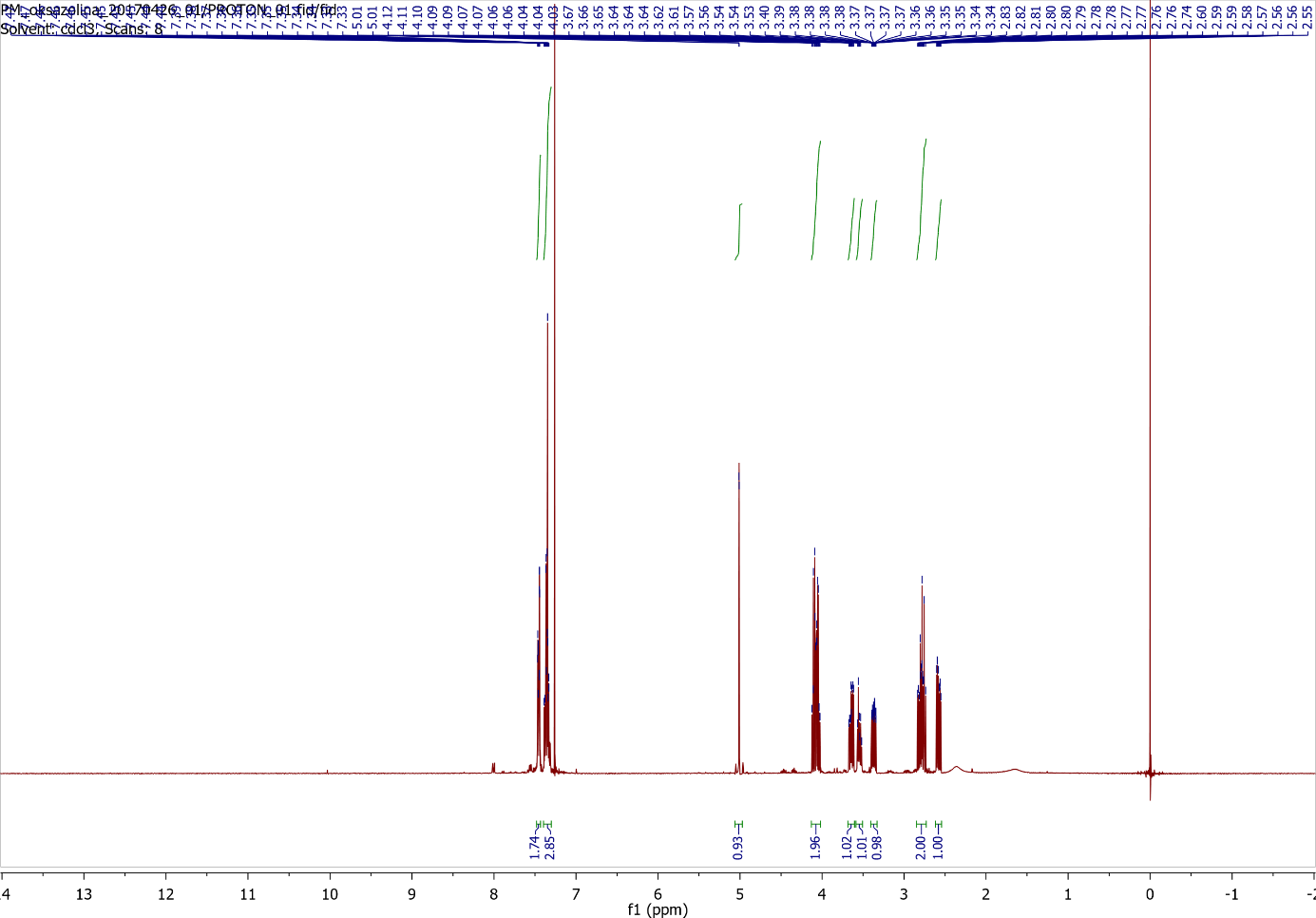


**Supplementary Figure 13.** ^1^H NMR spectrum of **N-(2-hydroxyethyl)-2-phenyloxazolidine.**


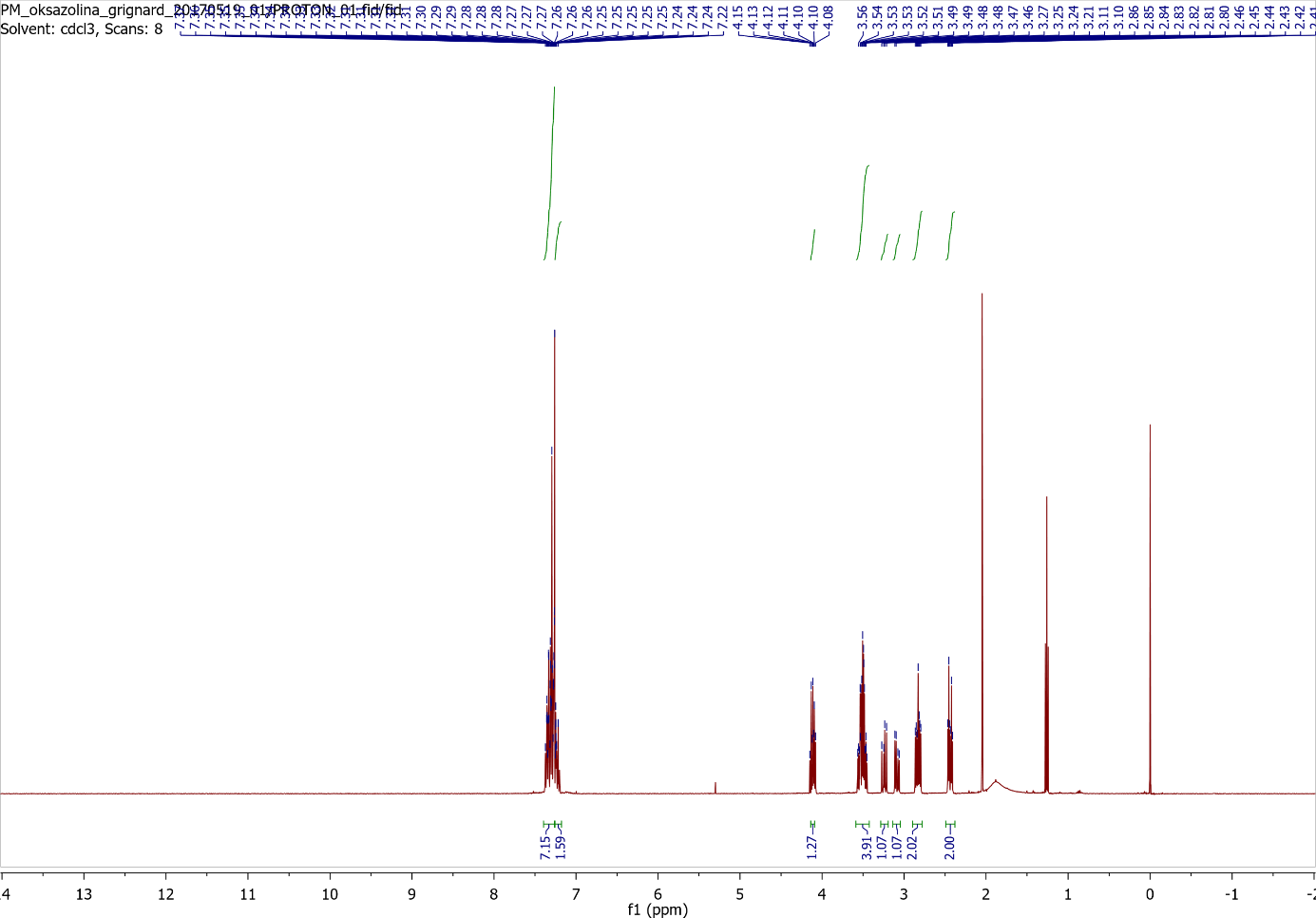


**Supplementary Figure 14.** ^1^H NMR spectrum of **N,N-bis(2-hydroxyethyl)-1,2-diphenylethylamine.**


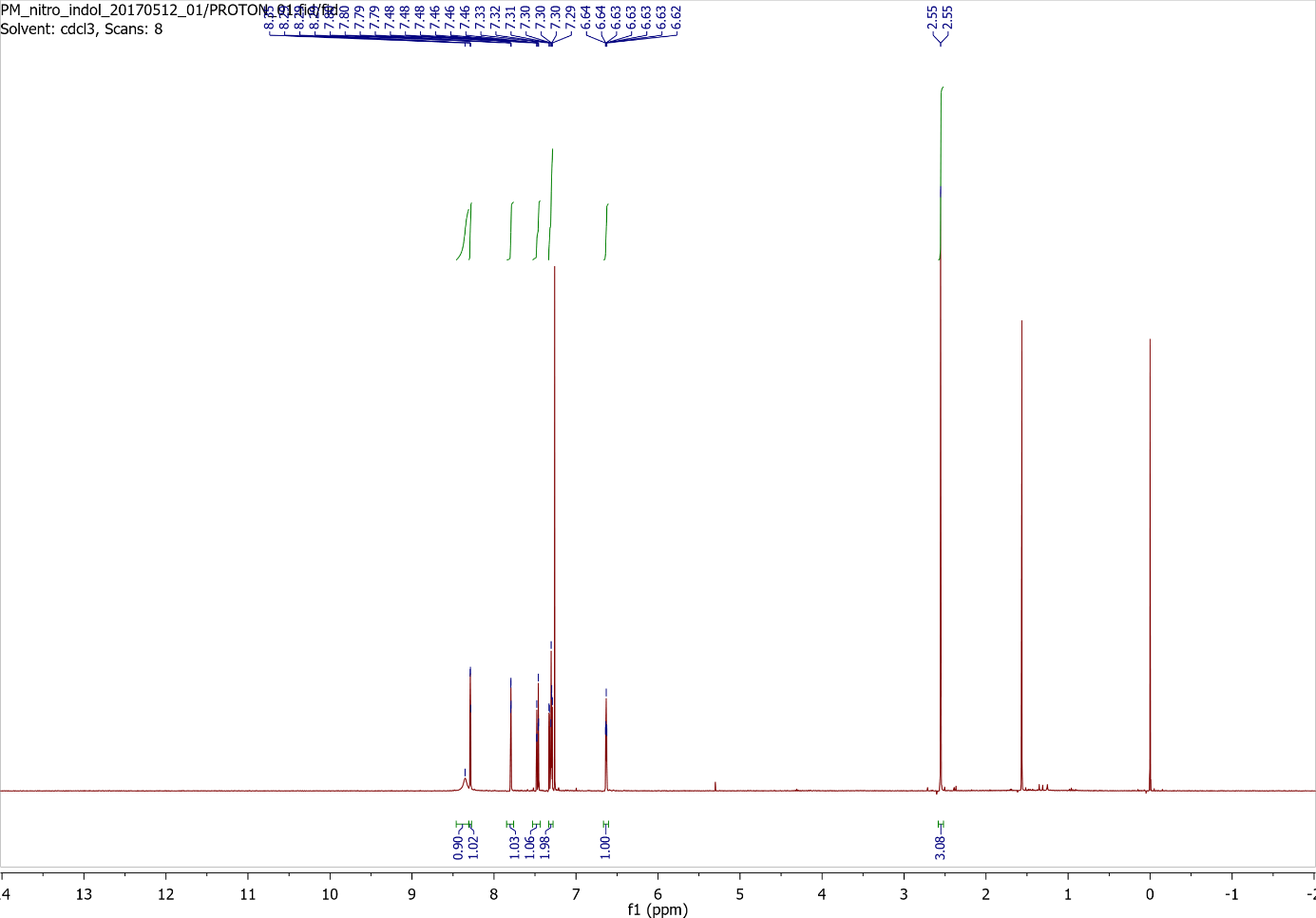


**Supplementary Figure 15.** ^1^H NMR spectrum of **5-(2-nitroprop-1-en-1-yl)indole.**


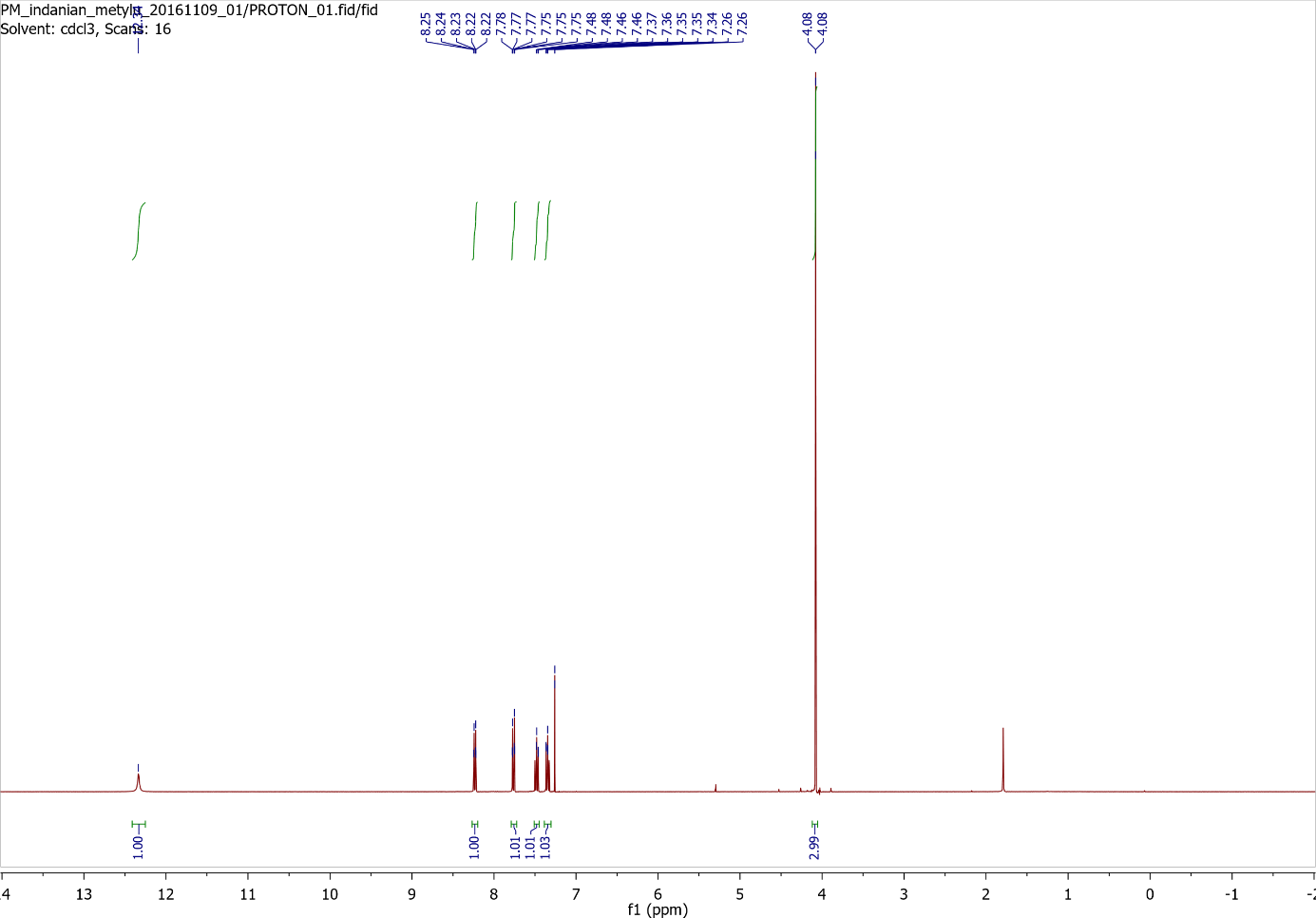


**Supplementary Figure 16.** ^1^H NMR spectrum of **Methyl 1H-indazole-3-carboxylate.**


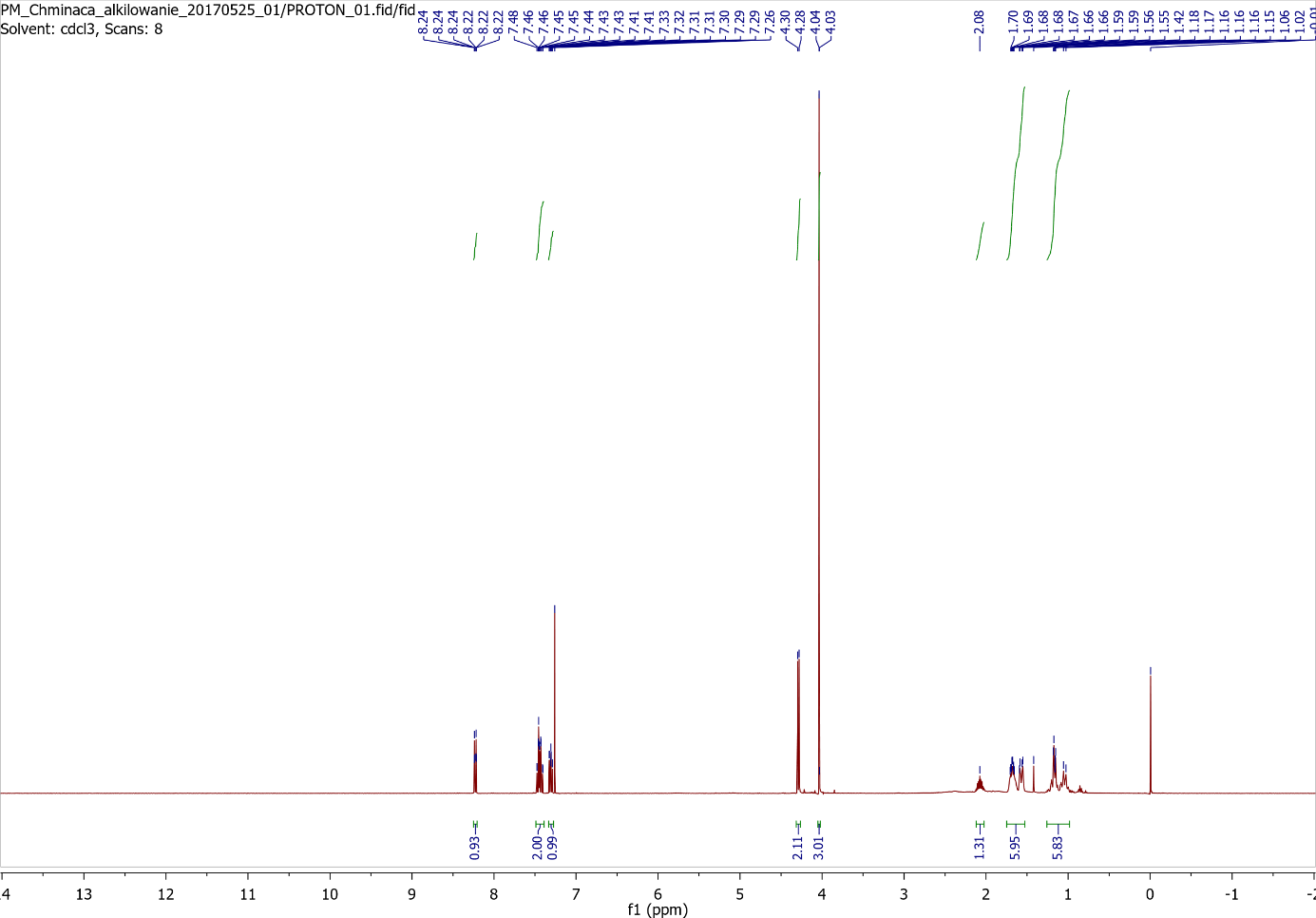


**Supplementary Figure 17.** ^1^H NMR spectrum of **Methyl 1-(cyclohexylmethyl)-indazole-3-carboxylate.**

**Mass spectra and chromatograms**

**Supplementary Figure 18.** Mass spectra for 5-IT (B)

5-IT: [M+H]^+^_theoret_ = 175.1230 Da

m/z = 158.0960 – in-source fragmentation of 5-IT

**Supplementary Figure 19.** Mass spectra for NM-2201 (B)

**NM-2201**:

[M+H]^+^_theoret_ = 376.1707

[M+K]^+^ _theoret_ = 414.1266

[M+Na]^+^_theoret_ = 398.1526

m/z = 232.1137 – in-source fragmentation of NM-2201

**Supplementary Figure 20.** Mass spectra for AB-CHMINACA (B)

AB-CHMINACA:

[M+H]^+^_teoret_ = 357.2285

[M+Na]^+^_teoret_ = 379.2104

**Supplementary Figure 21.** Mass spectra for UR-144 (B)

UR-144:

[M+H]^+^_theoret_ = 312.2322


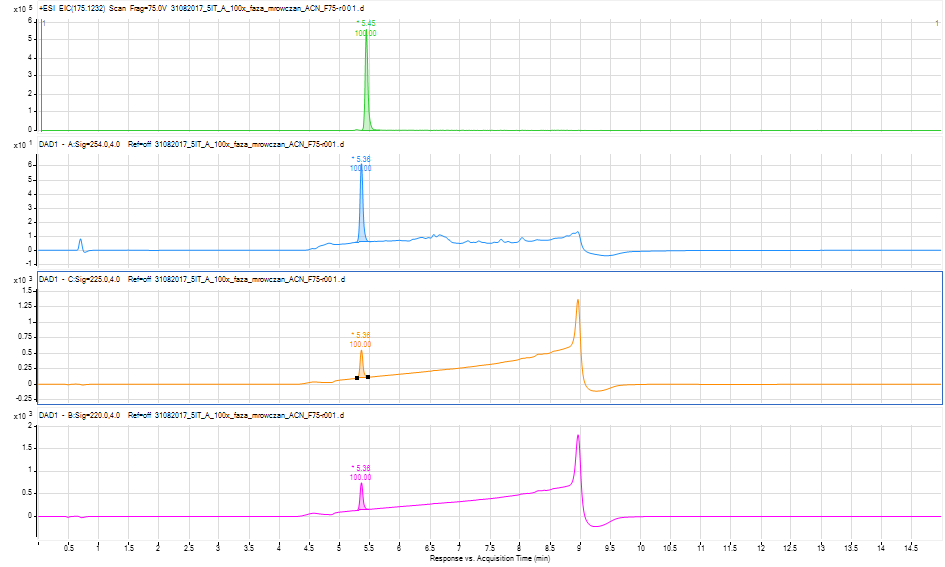


**Supplementary Figure 22.** Chromatogram of 5-IT from a laboratory scale (A). MS detection (extracted ion chromatogram) and UV-Vis at three wavelengths (254 nm, 230 nm and 295 nm).


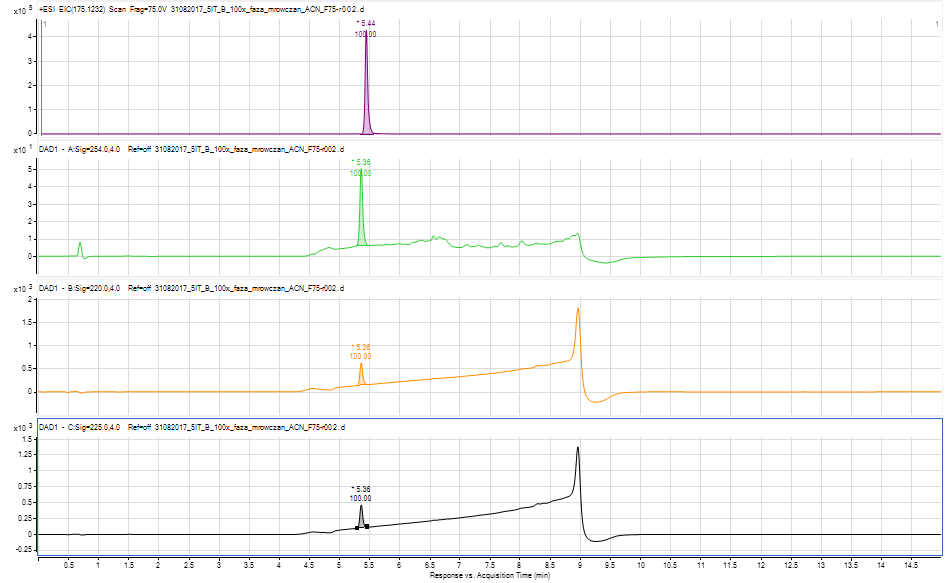


**Supplementary Figure 22.** Chromatogram of 5-IT synthesized on the semi-technical scale (B). MS detection (extracted ion chromatogram) and UV-Vis at three wavelengths (254 nm, 230 nm and 295 nm).

**Supplementary Figure 23.** Chromatogram of NM-2201 synthesized on the laboratory scale (A). MS detection (extracted ion chromatogram) and UV-Vis at three wavelengths (254 nm, 230 nm and 295 nm).

**Supplementary Figure 24.** Chromatogram of NM-2201 synthesized on the semi-technical scale (B). MS detection (extracted ion chromatogram) and UV-Vis at three wavelengths (254 nm, 230 nm and 295 nm).

**Supplementary Figure 25.** Chromatogram of AB-CHMINACA synthesized on the laboratory scale (A). MS detection (extracted ion chromatogram) and UV-Vis at three wavelengths (254 nm, 230 nm and 295 nm).

**Supplementary Figure 26.** Chromatogram of AB-CHMINACA synthesized on the semi-technical scale (B). MS detection (extracted ion chromatogram) and UV-Vis at three wavelengths (254 nm, 230 nm and 295 nm).

**Supplementary Figure 27.** Chromatogram of UR-144 synthesized on the laboratory scale (A). MS detection (extracted ion chromatogram) and UV-Vis at three wavelengths (254 nm, 230 nm and 295 nm).

**Supplementary Figure 28.** Chromatogram of UR-144 synthesized on the semi-technical scale (B). MS detection (extracted ion chromatogram) and UV-Vis at three wavelengths (254 nm, 230 nm and 295 nm).
